# Cardiomyocytes induced from hiPSCs by well-defined compounds have therapeutic potential in heart failure by secreting PDGF-BB

**DOI:** 10.1038/s41392-022-01045-4

**Published:** 2022-07-29

**Authors:** Hongmei Li, Fenfang Wu, Guangrui Huang, Di Wu, Ting Wang, Xiashuang Wang, Kai Wang, Yuyin Feng, Anlong Xu

**Affiliations:** 1 School of Life Science, Beijing University of Chinese Medicine, Beijing, China; 2 Dongzhimen Hospital, Beijing University of Chinese Medicine, Beijing, China; 3 Shenzhen Hospital, Beijing University of Chinese Medicine, Shenzhen, China; 4 College of Life Sciences, Sun Yat-Sen University, Guangzhou, China

**Keywords:** Stem-cell differentiation, Cardiology

## Abstract

Recent studies have suggested that transplant of hiPS-CMs is a promising approach for treating heart failure. However, the optimally clinical benefits have been hampered by the immature nature of the hiPS-CMs, and the hiPS-CMs-secreted proteins contributing to the repair of cardiomyocytes remain largely unidentified. Here, we established a saponin^+^ compound optimally induced system to generate hiPS-CMs with stable functional attributes in vitro and transplanted in heart failure mice. Our study showed enhanced therapeutic effects of optimally induced hiPS-CMs by attenuating cardiac remodeling and dysfunction, these beneficial effects were concomitant with reduced cardiomyocytes death and increased angiogenesis. Moreover, the optimally induced hiPS-CMs could gathering to the injured heart and secret an abundant PDGF-BB. The reparative effect of the optimally induced hiPS-CMs in the hypoxia-injured HCMs was mimicked by PDGF-BB but inhibited by PDGF-BB neutralizing antibody, which was accompanied by the changed expression of p-PI3K and p-Akt proteins. It is highly possible that the PI3K/Akt pathway is regulated by the PDGF-BB secreted from the compound induced hiPS-CMs to achieve a longer lasting myocardial repair effect compared with the standard induced hiPS-CMs. Taken together, our data strongly implicate that the compound induced hiPS-CMs promote the recovery of injured hearts via paracrine action. In this process, the paracrine factor PDGF-BB derived from the compound induced hiPS-CMs reduces isoproterenol-induced adverse cardiac remodeling, which is associated with improved cardiac function, and these effects are mediated by the PI3K/Akt pathway, suggesting that the optimally induced hiPS-CMs may serve as a new promising cell therapy for clinical applications.

## Introduction

Despite remarkable recent progress in the medical and surgical management of cardiac diseases, up to 10% of heart failure patients ultimately progress towards advanced and end-stage heart failure, which remains a major cause of death worldwide.^1,2^ The current gold-standard therapy in advanced heart failure is limited to mechanical cardiac support or heart transplantation, but most patients do not benefit from these therapies due to the eligible candidates far outnumber the available donor organs. As the heart lacks the regenerative potential to replace the dramatic cell loss that occurs during heart failure, numerous preclinical and clinical stem cell therapies have been intensively studied.^3–7^ However, the ideal cell type and the most effective way for those therapies are still not available.^8–10^

One therapeutic approach, which has several advantages, is the conversion of iPS cells into cardiomyocytes. By converting self-somatic cells into cardiomyocytes, one can reduce the risk of rejection since the cells are autologous.^11,12^ Studies in which hiPS-CMs or iPS-derived beating aggregates were injected showed positive increases in cardiac function and a reduced infarct size without any reports of teratoma formation.^13,14^ Despite these promising results, many lines of evidence indicate that under the conditions currently used, hiPS-CMs do not exhibit the morphological and functional characteristics of more mature cardiomyocytes, which is a barrier to their clinical use. The major technical problem in this approach is securing sufficient numbers of functional cardiomyocytes with high purity.

To solve these problems, here we reported a method for hiPS-CMs formation and functional maturation by adding our uniquely prepared saponin^+^ compound (mainly comprised of saponin and other well-defined small molecules) based on our previous research experience.^15^ Finally, we optimally generated the compound-induced hiPS-CMs that were highly consistent with the physiological characteristics of the late-stage of fetal heart development without the need of exogenous cells or genetic manipulation. We also compared the safety and effectiveness of the compound induced hiPS-CMs transplantation in a failure heart model, demonstrating a protective effect of the compound induced hiPS-CMs transplantation for heart failure therapy. In the further study of mechanism, we used sequencing technology to analyze mice hearts for the specific signaling pathway of the compound induced hiPS-CMs transplantation group, and the paracrine factors highly expressed in the supernatant of the hiPS-CMs culture were detected by cytokine protein array. From these experiments, PDGF-BB was identified as the key factor to regulate PI3K/Akt pathway to repair cardiomyocytes injury based on the mechanistic studies of in vitro hypoxia-injured HCMs model.

Collectively, our findings have revealed a paracrine factor PDGF-BB of the compound induced hiPS-CMs, which may contribute to the cardio-reparative effects of the hiPS-CMs transplantation and the underlying mechanisms of the therapy. These findings not only provide new insights into the mechanism of PDGF-BB in cardiomyocytes repair but also enrich our understanding of how the compound induced hiPS-CMs promote repair of the injured heart.

## Results

### Chinese medicine mixture increased the proportion of hiPS differentiated cardiomyocytes and drove their maturation

**Fig. 1:**
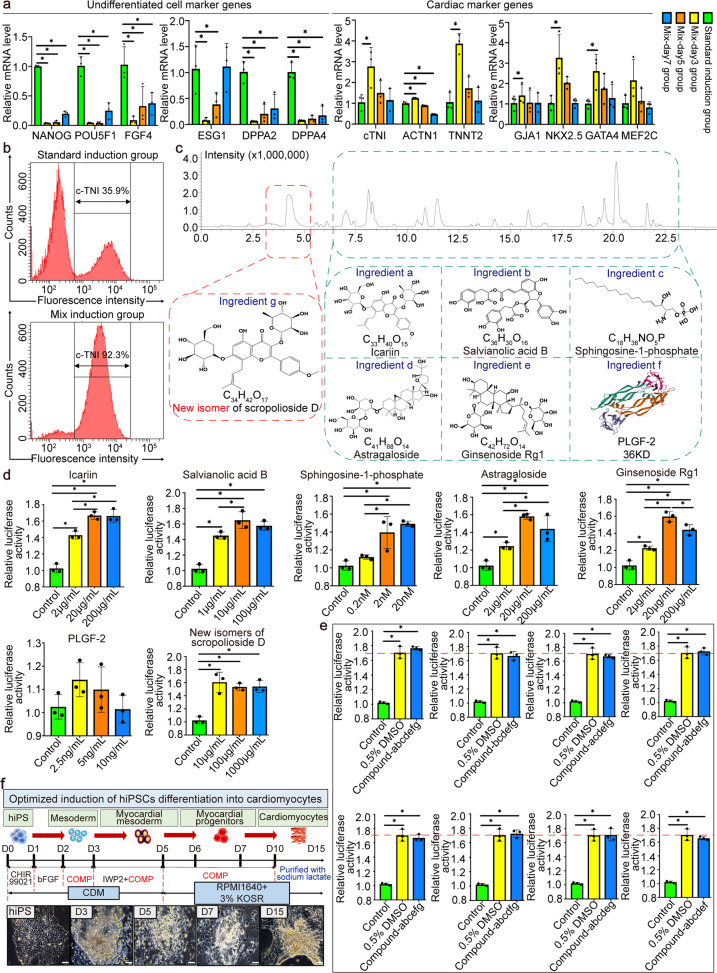
Developing the effective saponin^+^ compound derived from Chinese medicine mixture to optimally induce the differentiation of hiPS into cardiomyocytes. **a** Gene expression of unpurified hiPS-CMs optimally induced by Chinese medicine mixture at different intervention time points. Quantitative reverse transcription-polymerase chain reaction (RT-PCR) analyses of the expression levels of undifferentiated cell marker genes (NANOG, POU5F1, FGF4, ESG1, DPPA2, and DPPA4) and cardiac marker genes (cTNI, ACTN1, TNNT2, GJA1, NKX2.5, GATA4, and MEF2C). ^*^P < 0.05. **b** Flow cytometry was used to detect the positive rate of unpurified hiPS-CMs optimally induced by Chinese medicine mixture. Representative histogram of hiPS-CMs after differentiation showed the proportion of cTnI-expressing cells. **c** Identification of active ingredients in Chinese medicine mixture using LC–IT-TOF-MS. Core chemical structures of main ingredients a-g were shown. **d** Exploring the optimal concentration of each active ingredient in Chinese medicine mixture to induce P19 cells to differentiate into cardiomyocytes by drug screening system based on Myh6 promoter. The bar graphs represented averages and standard deviations, each performed in triplicate. The asterisk denoted statistical significance (*P* < 0.05). **e** Exploring the optimal combinations of active ingredients in Chinese medicine mixture to induce P19 cells to differentiate into cardiomyocytes by drug screening system based on Myh6 promoter. **f** Schematic representation of the differentiation procedure and sequential morphological changes (day 0–15) of hiPS differentiation into cardiomyocytes induced by effective saponin^+^ compound. Scale bars = 50 μm. hiPS, human-induced pluripotent stem cell

Before differentiation, we examined the pluripotency and karyotype of hiPSCs at day 0 and found that more than 95% of the cells highly expressed NANOG, OCT4, SOX2, and SSEA4 and had a normal karyotype (Supplementary Fig. 1). Based on the previous research methods,^16^ we have integrated a basic differentiation protocol called “standard induction scheme”. We first used CHIR99021 to inhibit the GSK3 β pathway, followed sequentially by bFGF and IWP2. After that, the cells were purified and maintained until they had further developed into beating cardiomyocytes (Movie S1). Next, we explored the best time for inducing the differentiation of cardiomyocytes by adding the Chinese medicine mixture into the standard induction scheme at different time points of the 3rd, 5th, and 7th day. The quantitative reverse transcription-polymerase chain reaction (RT-PCR) results showed that the expression of undifferentiated cell marker genes (NANOG, POU5F1, FGF4, ESG1, DPPA2, and DPPA4) was significantly lower, and the expression of cardiac marker genes (cTNI, ACTN1, TNNT2, GJA1, NKX2.5, GATA4, and MEF2C) was much higher in the Mix-day3 group than that in the other groups (Fig. 1a). Therefore, the 3rd day is the best time to start the Mix-induced differentiation. Then the differentiation rates were detected by flow cytometry, which showed that the proportions of c-TNI expressing cells in the Mix and standard induced group were 92.3% vs. 35.9% (Fig. 1b).

### Saponin^+^ compound with clear structure was developed from Chinese medicine mixture to achieve efficient and stable induction of hiPS into mature cardiomyocytes

LC–IT-TOF-MS was applied for the characterization of the process-related substances in Mix and seven main ingredients were determined. Among the identified ingredients (Fig. 1c), we could see that saponins were the main chemical constituents, 6 major ingredients were unambiguously identified as icariin (labeled as ingredient “a”), salvianolic acid B (labeled as ingredient “b”), sphingosine-1-phosphate (labeled as ingredient “c”), astragaloside (labeled as ingredient “d”), ginsenoside rg1 (labeled as ingredient “e”) and PLGF-2 (labeled as ingredient “f”). Furthermore, a new isomer of scropolioside D (labeled as ingredient “g”) was tentatively characterized in the present experiments.

Then, the optimal concentration of single ingredient to induce stem cells to differentiate into cardiomyocytes were evaluated by constructing recombinant luciferase reporter gene plasmid psiCheck-2-Myh6. The α-myosin heavy chain (α-MHC) encoded by Myh6 is a protein specific to myocardium, the higher the intensity of luciferase, the stronger the ability to differentiate cardiomyocytes. The luciferase reporter gene system was verified by positive drug 0.5% Dimethyl sulfoxide for inducing P19 cells to cardiomyocytes. Initially, the level of luciferase expression was less in control group, but after P19 cells in the presence of 20 μg/mL icariin, 10 μg/mL salvianolic acid B, 20 nM sphingosine-1-phosphate, 20 μg/mL astragaloside, 20 μg/mL ginsenoside rg1, 2.5 ng/mL PLGF-2, or 10 μg/mL new isomer of scropolioside D separately, the luciferase activity reached a maximum level (Fig. 1d). Furthermore, a full-component compound containing ingredient a-g (labeled as “Compound-abcdefg”) was tested in the first-round screening. The results showed that our optimized full-component compound had superior ability to induce P19 cells to differentiate into cardiomyocytes than that of the positive control group. In the second-round screening, seven compounds (labeled as “Compound-bcdefg”, “Compound-acdefg”, “Compound-abdefg”, “Compound-abcefg”, “Compound-abcdfg”, “Compound-abcdeg”, and “Compound-abcdef”) were prepared by removing ingredients one by one from the full-component compound, and statistical results displayed that dismantling any single ingredient would reduce the ability of full-component compound to induce cardiomyocyte differentiation to varying degrees (Fig. 1e). Thus, we prioritized full-component compound for the unique preparation of saponin^+^ compound. Further, we successfully verified and developed an optimized differentiation protocol in the hiPS system called “Compound induction scheme” by using the combined standard induction scheme and the full-component compound differentiation methods (Fig. 1f). By using this optimized induction scheme, we obtained stronger beating cardiomyocytes (Movie S2).

### Comparison of the stability of differentiated hiPS-CMs between the standard and compound induction schemes at micro and macroscale

A special microfluidic perfusion system has been developed to compare the stability of differentiated hiPS-CMs (Supplementary Fig. 3a, b). The presented results showed that the standard induced hiPS-CMs in the microfluidic device led to an obvious loss of hiPS-CMs relative to the culture dishes on the 4th and 5th day (20% area of cell loss is marked in red) and the start-up beating time was delayed to the 10th day (35% area of cell beating is marked in green). However, the compound induced hiPS-CMs in the microfluidic device showed stability of the differentiation process and more than 70% of the hiPS-CMs pulsated as early as the 9th day (Supplementary Fig. 3c).

### Saponin^+^ compound promoted the maturation of hiPS-CMs to the level of late-stage of fetal heart

**Fig. 2:**
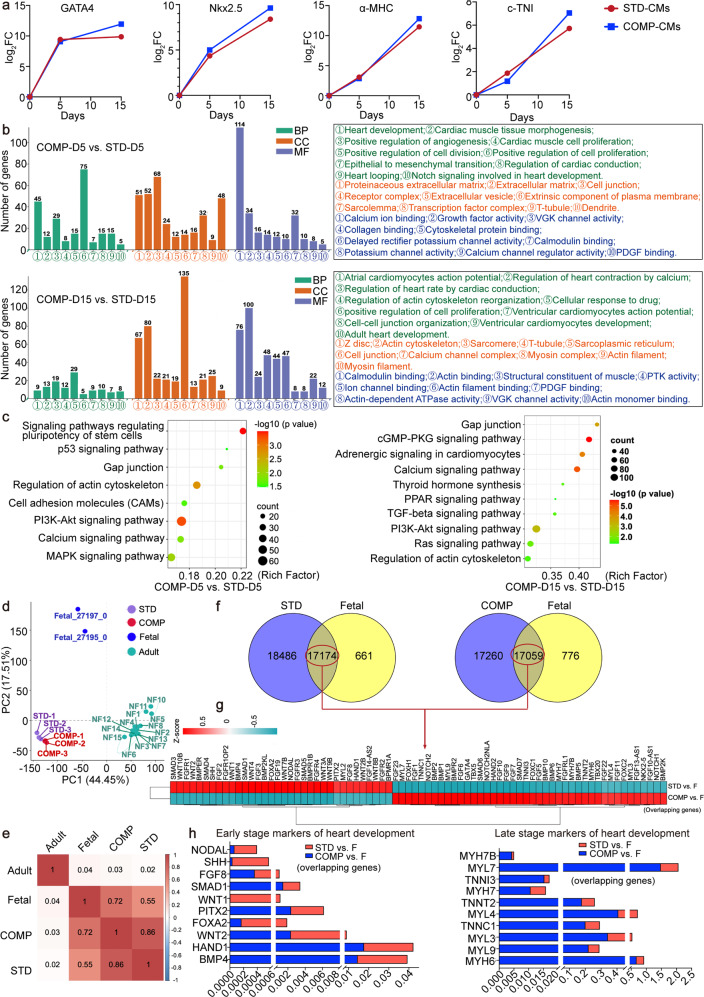
Gene expression profiling of the standard and compound induced hiPS-CMs from hiPSC lines UC (reprogrammed from urine cells). **a** RNA-Seq gene expression profiles of cardiomyocyte maturation markers GATA4, Nkx2.5, α-MHC, and c-TNI at day 0, 5, and 15 of the standard and compound-induced hiPS differentiation. Log2FC on the *y*-axis indicated the magnitude of gene differences between groups (STD-CMs, COMP-CMs) at two separate differentiation time points (day 5, day 15). Fold-change (FC) represented the differential fold-change in the differential mRNA between the two groups. Log2FC was obtained by taking the logarithm of FC at the base of 2. **b** GO functional classification of the standard and compound induced hiPS-CMs at day 5 and day 15. GO terms assigned to biological process, cellular component, and molecular functions. **c** KEGG functional classification of the standard and compound induced hiPS-CMs at day 5 and day 15. COMP-D5, the compound induced hiPS-CMs at day 5; STD-D5, the standard induced hiPS-CMs at day 5; COMP-D15, the compound induced hiPS-CMs at day 15; STD-D15, the standard induced hiPS-CMs at day 15. **d** Principal component analysis (PCA) was performed based on the shared genes of STD (D15), COMP (D15), Fetal and Adult. The first three principle components (PC) separated STD, COMP from Fetal and Adult. The first two axes accounted for 61.96% of variance. The first principal component (PC) explained 44.45% of the variance in the expression values and effectively separated the STD, COMP, Fetal, and Adult. The combination of the second and third principal components further separated the STD, COMP, Fetal, and Adult. Each symbol represented one sample. **e** Schematic representation of correlation matrix. Spearman correlation between different samples. The square color gradient effect showed the correlation coefficient. The correlation matrix values range from −1 to 1, representing either a completely negative or positive correlation, respectively. **f** Venn diagram showed the overlap between Fetal genes derived from GEO datasets and genes in STD (D15), or genes in COMP (D15). Fetal shared 17,174 and 17,059 genes with STD and COMP, respectively. **g** Heatmap showed the expression of the early and late-stage signature genes of heart development in overlap genes of “STD vs. Fetal” and “COMP vs. Fetal”, which indicated that the expression of the identified genes had different expression patterns in these types of samples. Expression is indicated as the z-score normalized. **h** Stacked column bar graphs depict the average gene expression at the early and late-stage of heart development. COMP expressed a larger proportion of late-stage genes of heart development than STD, showing more mature characteristics

We collected cells at various time points throughout the standard and compound differentiation protocol and performed RNA-seq to look for the transcriptomic expression of cardiomyocytes differentiation specific markers. It was important to note that the difference in expression of the GATA4, Nkx2.5, α-MHC, and c-TNI in the standard and compound induction groups began to widen from day 5, and the expression levels of cardiac early maturation markers GATA4 and Nkx2.5 in the compound induction group were higher between days 5 and 15, and its cardiac late maturation markers α-MHC and c-TNI increased rapidly and exceeded the standard induction group from the 8th day (Fig. 2a). GO analyses revealed that positive regulation of cell proliferation, cell junction and calcium ion binding were the highest terms in the compound induction group on the 5th day. On the 15th day of differentiation, cellular response to drug, cell junction and actin binding were the highest GO terms, respectively (Fig. 2b). Among the common uni-genes of KEGG analysis (*P* < 0.05), the highest representations were gap junction, PI3K-Akt pathway, calcium pathway and regulation of actin cytoskeleton both at day 5 and 15 after differentiation (Fig. 2c). Besides, we compared the RNA-seq data of purified standard induced hiPS-CMs (STD) and compound induced hiPS-CMs (COMP) on the 15th day of differentiation with the data on genes of fetal/adult hearts. It was found that the maturity of STD and COMP was closer to that of fetal heart (Fig. 2d, e). Furthermore, we compared STD and COMP with fetal cardiomyocytes respectively, it was demonstrated that COMP expressed more late-stage genes of fetal heart development, while STD expressed more early-stage genes (Fig. 2f–h), indicating that COMP has higher maturity than STD.

### Detection of the extracellular electrograms and intracellular Ca^2+^ measurement in the standard and compound induced hiPS-CMs

**Fig. 3:**
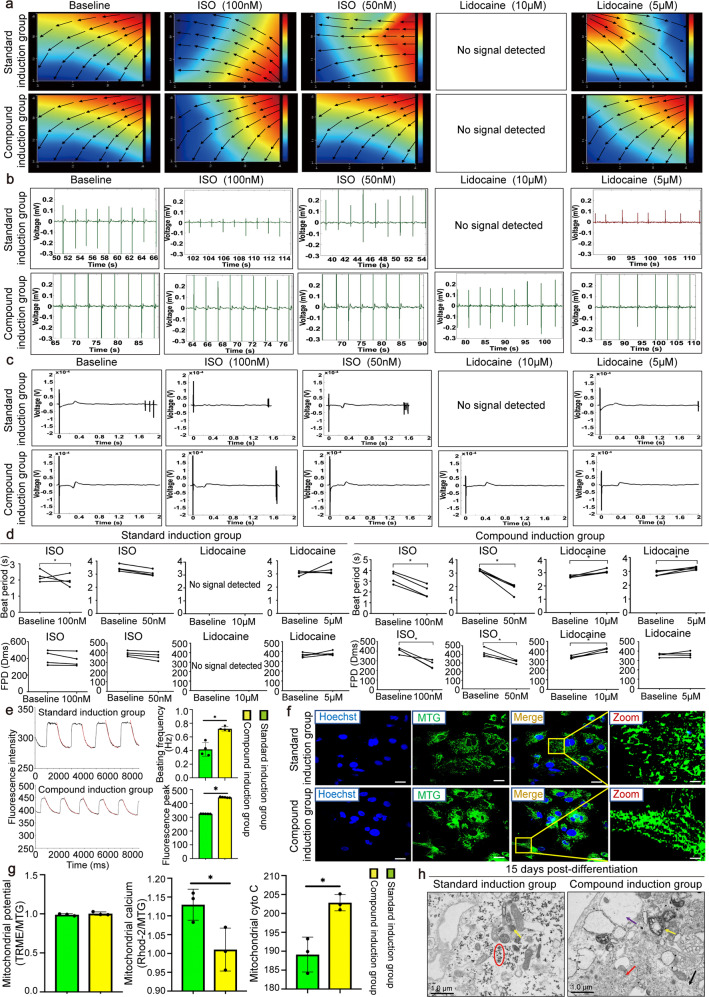
Comparison of extracellular electrograms and intracellular Ca2^+^ measurement as well as structure and functional maturity of the standard and compound induced hiPS-CMs from hiPSC lines UC (reprogrammed from urine cells). **a** An activation map serving as a visual representation of the activation sequence recorded by means of the MEA data acquisition system. The map activation time (the time duration between the first and last activations) is represented by the lower scale at the bottom of the map. The color strip below the map represents the color spectrum and its scaling according to time. Color coding: red-early; blue-late. **b** A representative display of electrograms recorded from the entire MEA array. Spontaneously beating cardiomyocytes were verified by extracellular electrograms recorded on the 3rd day after cell seeding and culture. **c** MEA micro matrix electrode system to detect the changes of field potential and phase waveform during depolarization/repolarization of hiPS-CMs. **d** Electrical properties of the cells were studied with MEA, which revealed the differences between the standard and compound induced hiPS-CMs group. FPD, field potential duration. **e** Calcium flux in hiPS-CMs at day 15 of the induction. Calcium transients were recorded at the basal condition. Images showed traces of calcium transients. Calcium transient frequency at the basal state (*n* = 4-5). Data were presented as the means ± SEM. ^*^P < 0.05. **f** Morphological and structural characteristics of mitochondria in living hiPS-CMs labeled with mitochondrial green fluorescent probe (MTG). Scale bars = 50 μm. **g** The fluorescence intensity of TMRE, Rhod-2 and cyto C staining were quantified using ImageJ, ^*^P < 0.05. **h** Ultrastructural examination of hiPS-CMs myofilament, microfilament, and mitochondria. Red arrow indicated myofilament. Black arrow indicated microfilament. Yellow arrows indicated mitochondria. Purple arrow indicated endoplasmic reticulum. Red circle indicated glycogen accumulation. Scale bars = 1 μm

We compared the electrophysiological functions of hiPS-CMs from hiPSC lines UC by MEA at the basal condition, after 100/50 nM ISO or 10/5 μM lidocaine treatment. The generated activation maps obtained from the basal condition from the compound and standard induced hiPS-CMs groups showed a general pattern of sequential activation, whereas the standard induction group after ISO treatment showed a disordered pattern with beat-to-beat variations and the propagation direction was completely reversed compared with baseline (Fig. 3a). Similar observations were consistently made in field potential, where the compound induction group could response with a stable field potential voltage after ISO/lidocaine stimulation (Fig. 3b). After activation by ISO, the depolarization and repolarization time of the compound induced hiPS-CMs were significantly shortened and regular in orders, while the standard induced hiPS-CMs showed irregular disorders. After different concentrations of lidocaine treatment, the compound induced hiPS-CMs showed stable dose-dependent response, while no signals or only residual weak signals were detected in the standard induced hiPS-CMs (Fig. 3c). Furthermore, the statistics on the field potential and repolarization quantification indicators showed that the compound induced hiPS-CMs had stronger drug sensitivity and tolerance, their electrophysiological conduction function was more stable compared with the standard induced hiPS-CMs (Fig. 3d). Moreover, the calcium transients showed significantly higher peak fluorescence and increased the frequency in the compound induced hiPS-CMs than in the standard induced hiPS-CMs group (Fig. 3e). Meanwhile, we induced the cardiomyocytes with another hiPSC lines BC and repeated above experiments and got the similar results (Supplementary Fig. 6a–f).

### More mature structure in the compound induced hiPS-CMs than that in the standard induced hiPS-CMs

In the standard induction group, MTG labeled mitochondrial appeared as short dots and rods. In the compound induction group, the mitochondrial morphology was relatively extended, in the form of a network and long lines (Fig. 3f). Mitochondrial membrane potential did not differ between the two groups under the basal conditions (Fig. 3g and Supplementary Fig. 5a), while the mitochondrial calcium was significantly lower in the compound induced hiPS-CMs (Fig. 3g and Supplementary Fig. 5b). Besides, there was a higher accumulation of cyto C in the mitochondria in the compound induced hiPS-CMs compared with the standard induced hiPS-CMs (*P* < 0.05), and no release of cyto C into the cytoplasm was observed in both groups (Fig. 3g and Supplementary Fig. 5c). Moreover, the formation of cytoskeleton such as the cell microfilaments and myofilaments could be seen under the electron microscope in compound induced hiPS-CMs (Fig. 3h), while no typical myofilament was found in standard induced hiPS-CMs, and the accumulation of glycogen was obvious. Additionally, we repeated above experiments using another hiPSC lines BC and obtained similar results (Supplementary Fig. 7a–f).

### In vivo imaging of hiPS-CMs labeled with CM-DiL in living mice

**Fig. 4:**
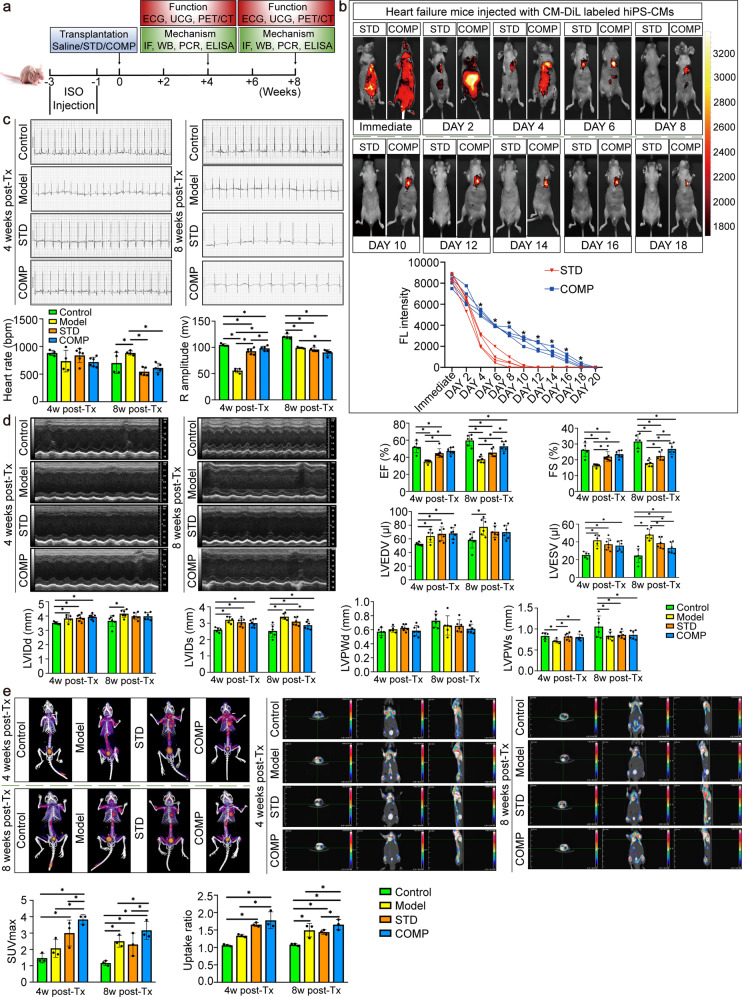
Effects of the standard and compound induced hiPS-CMs transplantation on cardiac function and myocardial energy metabolism. **a** Study protocol of the hiPS-CMs transplantation experiments in heart failure mice. **b** In vivo fluorescence imaging of mice demonstrated that the exogenously delivered compound induced hiPS-CMs labeled with CM-DiL could quickly gather to injured myocardium. Quantification of fluorescence intensity showed a longer cardiac aggregation effect in the compound induced hiPS-CMs than the standard induced hiPS-CMs transplantation group. Quantitative fluorescence intensity was shown on the right, *n* = 4 for each group. **c** Electrocardiogram (ECG) was performed 4 and 8 weeks after hiPS-CMs transplantation. *n* = 4–6 for each group. Representative traces from mice telemetric ECG recordings showed normal sinus rhythm. **d** Effects of the standard and compound induced hiPS-CMs on ventricular structure and function of heart failure mice at 4 and 8 weeks by echocardiography. Representative M-mode images from each group where the numbers used for calculations were derived. *n* = 5–7 for each group. EF, ejection fraction; FS, fractional shortening; LVEDV, left ventricular end-diastolic volume; LVESV, left ventricular end-systolic volume; LVIDd, left ventricular internal diastolic dimension; LVIDs, left ventricular internal systolic dimension; LVPWd, left ventricular posterior wall dimension in diastole; LVPWs, left ventricular posterior wall dimension in systole. **e** Myocardial metabolism was assessed via ^18^F-DPA-714 uptake by micro-PET/CT. *n* = 3 for each group. Representative 3D reconstruction images from global, transverse and other views from each group were shown. The higher glucose uptake level was shown as the increase in the intensity of white and red as indicated on the color scale bar shown in each image. Calculation of SUVmax and uptake ratio showed that the compound induced hiPS-CMs transplantation group had a significantly increased glucose uptake level. STD, standard induced hiPS-CMs transplantation group; COMP, compound induced hiPS-CMs transplantation group

We transplanted the standard and compound induced hiPS-CMs to mice 1 week after successful modeling (Fig. 4a). Then noninvasive imaging was performed to assess homing and bio-distribution. The higher imaging signals in the COMP indicated that more hiPS-CMs were gathering to the injured hearts compared with STD. The duration of the fluorescent signal activity in the local heart was 18 days and the relative signal decay time was late in the COMP, much longer than the STD (Fig. 4b).

### Effect of hiPS-CMs transplantation on electrophysiological function of heart failure

ECG of hiPS-CMs transplanted mice detection at different time points revealed a significantly shortened R wave amplitude. Obvious recovery of R wave amplitude occurred within the first 4 weeks, but it shortened again after 8 weeks. The incidence of sinus bradycardia was highest in the 8th week after hiPS-CMs transplantation. No significant difference was found in heart rate or R wave amplitude between compound and standard induced hiPS-CMs transplantation groups in the 8th week (Fig. 4c).

### Improvement in global cardiac performance after hiPS-CMs transplantation

Serial standard transthoracic echocardiography was performed before, and 4 and 8 weeks after the hiPS-CMs transplantation (Fig. 4d). Both of the standard and compound induced hiPS-CMs transplantation therapies showed improvement in EF compared with the model group. Subsequently, the EF at the 8th week was significantly greater in the compound induced hiPS-CMs group than that in the standard induced hiPS-CMs group (52.79 ± 5.65% vs. 45.41 ± 4.58%, *P* < 0.05). FS was also significantly greater in the compound induced hiPS-CMs group. Furthermore, indexes reflecting myocardial remodeling such as LVESV and LVIDs at the 8th week in the compound induced hiPS-CMs group were smaller than those in the standard induced hiPS-CMs group. These parameters indicated that the compound induced hiPS-CMs had stronger effects on improving myocardial performance than the standard induced hiPS-CMs.

### Accumulation of glucose uptake after the compound induced hiPS-CMs transplantation improved myocardial energy reconstruction

Micro-PET/CT was used to analyze the level of glucose uptake after hiPS-CMs transplantation (Fig. 4e). From the 3D composition results, the heart failure mice could provide an energy supply to the failing myocardium by enhancing glucose metabolism. Compared with the heart failure mice, the myocardial glucose metabolism was significantly increased after hiPS-CMs treatment, while compound induced hiPS-CMs promoted glucose metabolism more obviously than the standard induced hiPS-CMs. Then, we performed 2D scanning at multiple angles to quantify the glucose uptake. The SUVmax and uptake ratio were significantly higher, especially at 8 weeks, in the compound induced hiPS-CMs group than that in the standard induced hiPS-CMs group, the control group or the model group (*P* < 0.05).

### Safety and efficacy of the compound induced hiPS-CMs transplantation confirmed by pathological and ultrastructural changes

**Fig. 5:**
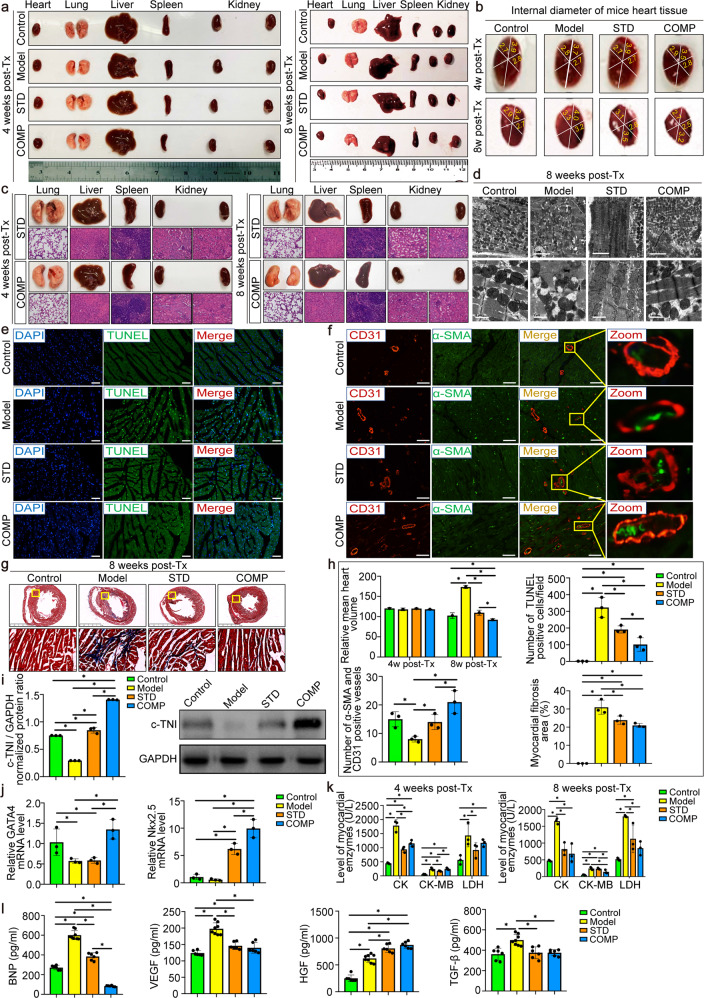
Effects of the compound induced hiPS-CMs on cardiac structure and expression of peripheral blood secretion factors after 8 weeks of transplantation. **a** Preliminary safety evaluation by macroscopic comparison of important organs morphology. **b** Diameter analysis at 8 weeks post-transplantation showed significantly smaller sizes of the heart in the compound induced hiPS-CMs transplantation group than in the heart failure model group. **c** Neither macroscopic nor microscopic analysis revealed any evidence of tumor formation at 4 and 8 weeks after hiPS-CMs transplantation. **d** Ultrastructural changes in cardiomyocytes of control, model and hiPS-CMs transplanted groups were revealed by transmission electron microscope analysis at low (scale bar: 5 μm) and high (scale bar: 1 μm) magnifications. **e** TUNEL assay was performed to detect the myocardial apoptosis. Apoptotic nuclei were stained green, and normal nuclei were stained blue. Scale bar: 50 μm. **f** Microvascular neo-angiogenesis at 8 weeks post hiPS-CMs transplantation. Immunofluorescent staining for micro-vessels positive for α-smooth muscle actin (α-SMA) and CD31 in the left ventricle. Scale bar: 200 μm. **g** Representative photographs of myocardial fibrosis, which were determined by Masson’s trichrome staining. The blue color represented the distribution of collagens. **h** Statistical analysis of myocardial apoptosis, microvascular neo-angiogenesis and fibrosis staining were quantified using ImageJ. **i** The expression of c-TNI protein was analyzed by Western blotting and normalized to GAPDH. **j** Real-time PCR was used to assess cardiac transcription factor GATA4 and Nkx2.5 mRNA levels in the heart. **k** Expression of myocardial injury markers in peripheral blood. CK, CK-MB and LDH are very sensitive and specific indicators of damage to the myocardium. **l** Expression of secretory factors in peripheral blood. BNP is a factor released into the blood by injured myocardium, and its concentration changes reflect the severity of heart failure. VEGF, HGF and TGF-β are feedback-activated factors during ventricular pathological remodeling in failing myocardium. *n* = 3-4 mice per group and * indicated that *P* < 0.05. STD, standard induced hiPS-CMs transplantation group; COMP, compound induced hiPS-CMs transplantation group

To evaluate the safety and efficacy of hiPS-CMs transplantation, we removed the heart, liver, spleen, lung and kidney for morphological comparisons and observed significant changes in cardiac morphology at 4 and 8 weeks after hiPS-CMs transplantation (Fig. 5a). Then we measured the size of the heart and found that after 8 weeks of heart failure, the heart began to expand significantly, and the enlarged heart after hiPS-CMs transplantation had different degrees of retraction. Compared with the standard induced hiPS-CMs group, the heart in the compound induced hiPS-CMs group recovered more obviously, but the above characteristic morphological changes were not obvious at 4 weeks (Fig. 5b, h). In addition, no teratoma formation was found in all major organs (Fig. 5c) and no specific liver or kidney functional damage was observed (Table 1). Then, we examined the ultrastructure of the myocardium in each group. Eight weeks after the hiPS-CMs transplantation, the myofibrils of the myocardium recovered obviously, the Z-line structure tended to be complete, and the myofilaments were closely arranged, but there was still slight swelling and vacuolation of the mitochondria in the standard induced hiPS-CMs group, and disappearance of the mitochondrial ridges persisted in the compound induced hiPS-CMs group (Fig. 5d).

**Table 1:** Biochemical safety index detection post-transplantation

			4 weeks post-transplantation		8 weeks post-transplantation	
Biochemical index	Control	Model	Standard induced hiPS-CMs	Compound induced hiPS-CMs	Standard induced hiPS-CMs	Compound induced hiPS-CMs
ALT (U/L)	52.167 ± 5.193	59.667 ± 7.024	40.667 ± 4.619	72.250 ± 29.285	38.667 ± 3.055^#*^	37.667 ± 3.055^#*^
AST (U/L)	150.17 ± 29.28	193.00 ± 22.52	121.00 ± 15.59^*^	207.25 ± 48.40^#^	133.33 ± 23.18^*^	128.33 ± 22.14^*^
TP (g/L)	53.250 ± 2.449	51.767 ± 0.681	50.967 ± 2.237	55.225 ± 1.212^*^	51.133 ± 0.808	52.467 ± 4.267
ALB (g/L)	34.533 ± 1.357	32.733 ± 0.31^#^	32.967 ± 0.950	34.900 ± 0.812^*^	32.400 ± 0.600	32.700 ± 2.551
GLOB (g/L)	18.717 ± 2.145	19.033 ± 0.451	18.000 ± 1.400	20.325 ± 1.340	18.533 ± 0.503	19.767 ± 1.779
A/G	1.862 ± 0.209	1.720 ± 0.035	1.790 ± 0.177	1.725 ± 0.130	1.7033 ± 0.100	1.657 ± 0.055
TBIL (μmol/L)	0.8833 ± 0.183	1.1333 ± 0.611	1.1333 ± 0.153	0.7750 ± 0.171	0.900 ± 0.1000	0.7667 ± 0.058
DBIL (μmol/L)	0.2000 ± 0.063	0.3000 ± 0.265	0.3000 ± 0.100	0.1250 ± 0.126	0.1667 ± 0.058	0.0667 ± 0.058^*^
IBIL (μmol/L)	0.6833 ± 0.183	0.8333 ± 0.351	0.8000 ± 0.100	0.7000 ± 0.082	0.7000 ± 0.100	0.7000 ± 0.000
GLU (mmol/L)	8.8250 ± 1.046	7.7733 ± 0.834	9.5567 ± 0.5705	7.2325 ± 1.3666^#^	8.070 ± 0.9453	8.2333 ± 1.052
UREA (mmol/L)	7.0167 ± 0.627	6.6333 ± 0.603	6.6333 ± 0.850	7.7000 ± 0.8485	6.3000 ± 0.557	6.3667 ± 0.7234
UA (mmol/L)	195.67 ± 30.12	129.33 ± 75.87	158.33 ± 10.79	169.00 ± 46.195	161.67 ± 9.074	156.00 ± 21.703
CREA (μmol/L)	7.5000 ± 1.761	5.333 ± 0.577^#^	9.0000 ± 1.732^*^	8.5000 ± 0.5773^*^	13.333 ± 8.505^*^	11.6667 ± 3.7859

Data were the means ± SEM

^#^*P* < 0.05 compared with corresponding control value

^*^P < 0.05 compared with corresponding model value

*ALT* alanine aminotransferase, *AST* aspartate aminotransferase, *TP* total protein, *ALB* albumin, *GLOB* globulin, *A/G* albumin and globulin ratio, *TBIL* total bilirubin, *DBIL* direct bilirubin, *IBIL* indirect bilirubin, *CK* creatine kinase, *CK-MB* creatine kinase isoenzyme, *LDH* lactate dehydrogenase, *GLU* glucose, *UREA* blood urea nitrogen, *UA* uric acid, *CREA* creatinine

### Attenuation of cardiac cell death and fibrosis after the compound induced hiPS-CMs transplantation

Cardiac apoptosis was examined by the TUNEL assay using frozen sections of myocardial tissue. In contrast with the model group, the number of apoptotic cells decreased significantly after compound induced hiPS-CMs transplantation (Fig. 5e and Supplementary Fig. 8a), while this phenomenon was not observed in the standard induced hiPS-CMs transplantation group at 4 weeks. After quantitative statistical analysis of the TUNEL-positive cells, it was found that the number of apoptotic cells in the compound induced hiPS-CMs group decreased more than that of the standard induced hiPS-CMs group at 4 and 8 weeks (Fig. 5h and Supplementary Fig. 8d). Next, we examined the collagen volume fraction in the LV myocardium following Masson staining. The collagen volume fraction was significantly reduced in the compound induced hiPS-CMs treatment group compared with the model mice after 4 and 8 weeks of transplantation (Fig. 5g, h). However, 4 weeks of compound and standard induced hiPS-CMs transplantation couldn’t obviously attenuate cardiac fibrosis, but significant statistical differences between the two groups of mice were seen (Supplementary Fig. 8c, d).

### Compound induced hiPS-CMs transplantation enhanced neovascularization to increase myocardial blood supply

The micro-vascularity was demonstrated by α-smooth muscle actin and CD31 co-staining. We could see that the vascular intima in the control group was mostly continuous, while the microvascular wall in the model group was broken. After standard induced hiPS-CMs transplantation, the continuity of the microvascular wall was partially repaired but not ideal. In contrast, after the compound induced hiPS-CMs treatment, the continuity of the microvascular wall was further repaired (Fig. 5f and Supplementary Fig. 8b). The statistical analysis revealed that the number of micro-vessels increased significantly after 8 weeks of hiPS-CMs transplantation compared with the model group and there were significant differences between the standard and compound induced hiPS-CMs groups (Fig. 5h). However, the density of micro-vessels after 4 weeks of hiPS-CMs transplantation did not differ statistically from the model group (Supplementary Fig. 8d). In addition, we found that the expression of c-TNI in mice heart increased after 8 weeks of hiPS-CMs transplantation, and the compound induced hiPS-CMs group increased the most (Fig. 5i). In contrast, there was no obviously change in c-TNI expression at 4 weeks (Supplementary Fig. 8e).

### Damaged myocardium repaired by activating myocardial transcription factors and neovascularization-related plasma factors after hiPS-CMs transplantation

We examined the expression levels of myocardial transcription factors in the myocardium by RT-PCR. The expression of GATA4 was significantly greater in the compound induced hiPS-CMs group than those in the model group at 4 and 8 weeks. Besides, the gene expression of Nkx2.5 increased in the compound induced hiPS-CMs group at 8 weeks, while no difference was found at 4 weeks (Fig. 5j and Supplementary Fig. 8f). Then plasma was isolated from mouse blood to detect the expression of BNP, CK, CK-MB, and LDH. At 4 weeks after transplantation, CK, CK-MB, and LDH were significantly reduced in the standard induced hiPS-CMs group but were not significantly lower in the compound induced hiPS-CMs group. At 8 weeks, the compound induced hiPS-CMs group had significantly reduced CK, CK-MB and LDH, but with no significant difference compared with the standard induced hiPS-CMs group (Fig. 5k). ELISA tests found that the expression of VEGF, HGF and TGF-β in the model mice showed a feedback increase compared with the control group. Eight weeks after the hiPS-CMs transplantation, BNP, VEGF, and TGF-β decreased significantly but there was an increase in HGF expression (Fig. 5l).

### The apoptosis-related PI3K/Akt pathway specifically regulated by the compound induced hiPS-CMs was the key to have better efficacy on myocardial repair

**Fig. 6:**
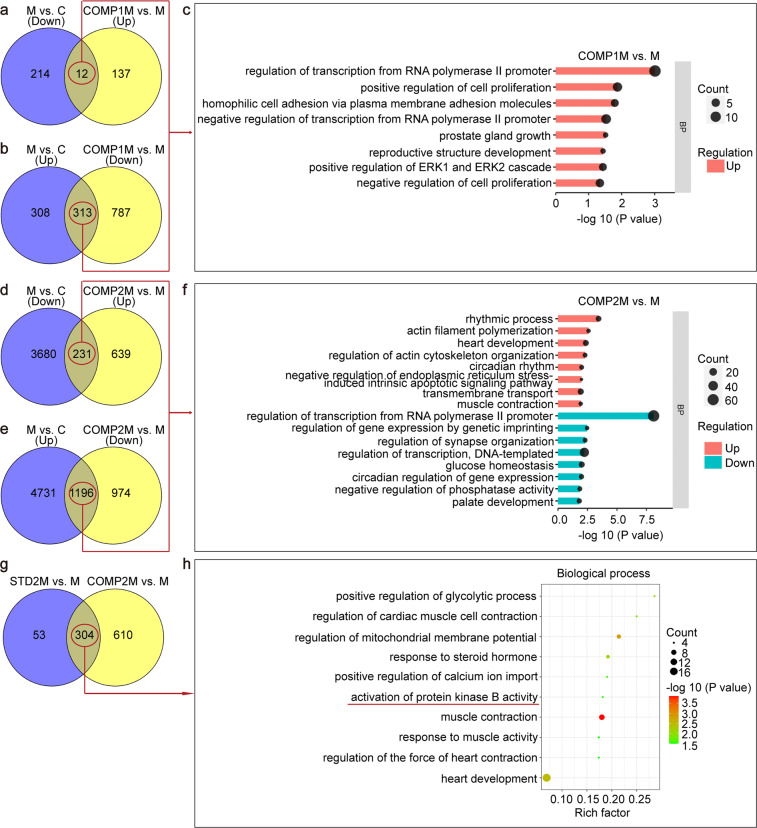
Gene expression profiling of the heart by RNA-seq after hiPS-CMs transplantation treatment. **a** Venn diagram showing that the upregulated differentially expressed genes (DEGs) of “COMP1M vs. M” group overlap with the downregulated DEGs of “M vs. C” group, for a total of 12 overlapping genes. **b** Venn diagram showing the intersection of upregulated DEGs in the “M vs. C” group and the downregulated DEGs in the “COMP1M vs. M” group, with a total of 313 intersecting genes. **c** Gene ontology (GO) enrichment analysis of the 12 and 313 DEGs obtained above. The biological pathways that were enriched among genes were ranked based on –log10(*P*-value). Enrichment was performed using the DAVID database. The eight of the most significantly enriched biological process GO terms were shown (*P* < 0.05). **d** Venn diagram showing 231 overlapping genes between the downregulated DEGs of “M vs. C” and upregulated DEGs of “COMP2M vs. M” groups. **e** Venn diagram of upregulated DEGs of “M vs. C” group compared with downregulated DEGs of “COMP2M vs. M” group. A total of 1196 shared genes. **f** Enrichment of GO biological processes identified from 231 and 1196 shared DEGs, respectively. **g** Venn dia**g**ram showing “STD2M vs. M” and “COMP2M vs. M” shared DEGs, with a total of 304 shared DEGs in both groups. **h** The most differentially overrepresented biological processes in “COMP2M vs. M” compared with “STD2M vs. M” were displayed. Three mice per group. “Count” means that the enriched DEGs numbers in the pathway. “RichFactor” represents the number of DEGs relative to total number of genes in the pathway, with larger RichFactors indicating greater enrichment. COMP1M, one month after the saponin^+^ compound induced hiPS-CMs transplantation treatment group; COMP2M, two months after the saponin^+^ compound induced hiPS-CMs transplantation treatment group; STD2M, two months after the standard induced hiPS-CMs transplantation treatment group; M, heart failure model group; C, control group. COMP1M vs. M, DEGs in the saponin^+^ compound induced hiPS-CMs transplantation group treated for 1 month compared with the model group; COMP2M vs. M, DEGs in the saponin^+^ compound induced hiPS-CMs transplantation group treated for 2 months compared with the model group; STD2M vs. M, DEGs in the standard induced hiPS-CMs transplantation group treated for 2 months compared with the model group; M vs. C, DEGs in the model group compared with the control group

RNA-sequence analysis of the mouse myocardium at 4 and 8 weeks after the standard and compound induced hiPS-CMs transplantation showed that COMP1M and COMP2M shared a common enrichment in the upregulated overlapping DEG involved in negative regulation of endoplasmic reticulum stress induced intrinsic apoptotic signaling pathway, largely reversed apoptosis effect in the model group (Fig. 6a–f). To clarify whether COMP2M also have the advantage of anti-apoptosis in contrast with STD2M, we constructed Venn diagrams with gene sets that were significantly altered (*P* < 0.05) in one pairwise comparison between “STD2M vs. M” and “COMP2M vs. M”, further identified 304 overlapping DEGs (Fig. 6g). Then GO analysis was conducted, which revealed that calcium ion import, muscle contraction and the activation of protein kinase B activity were highly enriched (Fig. 6h). Of these, protein kinase B is most closely associated with the above-mentioned regulation of apoptosis. The activation of PKB promoted myocardial cell repair was basically achieved by activating the PI3K/Akt pathway, which demonstrated that the compound induced hiPS-CMs transplantation effects were associated with anti-apoptosis responses such as activation of PI3K/Akt pathway.

### PDGF-BB secreted from the compound induced hiPS-CMs rescued the cell model from hypoxia-induced human cardiac myocytes injury

**Fig. 7:**
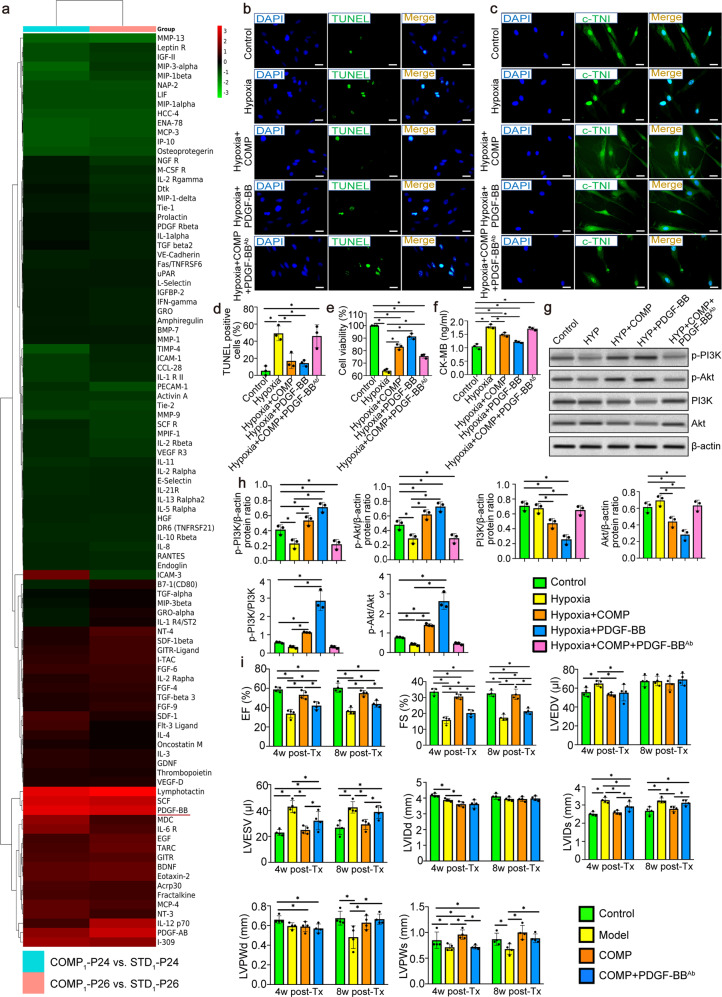
PDGF-BB secreted from the compound induced hiPS-CMs from hiPSC lines UC (reprogrammed from urine cells) rescued the hypoxia-triggered injury model of HCMs by regulating PI3K/Akt pathway in vitro and played a key role in the recovery of cardiac function in vivo. **a** Heat map demonstrated relative expressions of biomarkers from screening cytokine antibody array in the supernatant of the compound and standard induced hiPS-CMs derived from two different generations of hiPS cells (passage 24, 26). The upregulated cytokines were shown in red and downregulated cytokines in green. **b** Representative photomicrographs of TUNEL (green) and DAPI (blue) -stained HCMs were shown. Scale bars = 50 μm. **c** Changes of myocardial specific protein c-TNI expression level in hypoxia-injured HCMs was detected by immunofluorescence assay. Scale bars = 50 μm. **d** Quantification of IF staining for TUNEL^+^ HCMs showed that PDGF-BB in the conditioned medium of the compound induced hiPS-CMs was the core cytokine that inhibited the apoptosis of HCMs. **e** Quantitative analysis by CCK-8 assay of cell viability. Values were expressed as the mean ± SD of three independent experiments. **f** The protein expression of CK-MB in the extracellular medium of hypoxia-injured HCMs was detected by ELISA. **g** PI3K, p-PI3K, Akt, p-Akt and β-actin levels of hypoxia-injured HCMs in Control, HYP, HYP + COMP, HYP + PDGF-BB and HYP + COMP + PDGF-BB^Ab^ groups were analyzed by Western blot. **h** The protein expression levels were normalized to those of β-actin. All data shown as mean ± SD, ^*^P < 0.05. **i** PDGF-BB^Ab^ counteracted most of the protective effects of compound induced hiPS-CMs on cardiac function in vivo at 4 and 8 weeks by echocardiography. *n* = 4 for each group. HCMs, human cardiac myocytes; HYP: Hypoxia; COMP, the conditioned medium of the compound induced hiPS-CMs; PDGF-BB, platelet-derived-growth-factor-BB; PDGF-BB^Ab^, neutralizing antibody against PDGF-BB; c-TNI: cardiac Troponin I; COMP-P24, the conditioned medium from cardiomyocytes induced by the 24th generation of hiPS by the compound induction scheme; COMP-P26, the conditioned medium from cardiomyocytes induced by the 26th generation of hiPS by the compound induction scheme; STD-P24, the conditioned medium from cardiomyocytes induced by the 24th generation of hiPS by the standard induction scheme; STD-P26, the conditioned medium from cardiomyocytes induced by the 26th generation of hiPS by the standard induction scheme; p-PI3K: phospho-phosphatidylinositol 3-kinase; PI3K: phosphatidylinositol 3-kinase; p-Akt: phospho- protein kinase B; Akt: protein kinase B

Cytokine array analysis was performed to detect the expression of 174 cytokines using conditioned medium from cardiomyocytes induced by two different generations of hiPSC lines UC (passage 24 and 26). Cluster analysis and Heatmap of the common differential proteins in the factors from hiPS-CMs showed that there were differences in the expression of factors secreted by standard and compound induced hiPS-CMs (Fig. 7a). Three factors including PDGF-BB, lymphotactin and SCF, which were shown as specific high expression factors in the compound induced hiPS-CMs were noticed. Among these factors, PDGF-BB could protect various cell types from apoptotic cell death. Linked to our results of animal experiments in vivo, we chose PDGF-BB as the entry point in our subsequent research of anti-apoptotic mechanism.

To determine whether PDGF-BB could really restore damaged myocardiocytes, we analyzed the cell apoptosis, cell viability, HCMs-specific cytoplasmic marker protein, and injury marker of HCMs supernatant to assess the restorative effects with conditioned medium of the compound induced hiPS-CMs (COMP medium), as well as the repair effect of adding PDGF-BB alone. After quantitative analysis of the TUNEL staining (Fig. 7b, d), the percent apoptotic nuclei was significantly reduced in Hypoxia+COMP and Hypoxia+PDGF-BB groups compared with Hypoxia model group. In terms of cell viability, CCK-8 assay demonstrated that Hypoxia model mimic inhibited HCMs viability and this inhibition effect was largely counteracted by the COMP medium and PDGF-BB (Fig. 7e). We then detected the expression of c-TNI in HCMs and found that c-TNI was largely decomposed when HCMs were damaged by hypoxia and this process was also reversed by COMP medium and PDGF-BB (Fig. 7c). Similarly, the amount of CK-MB released extracellularly in the Hypoxia+COMP and Hypoxia+PDGF-BB groups exhibited to be basically restored (Fig. 7f). These results suggested that the effect of COMP medium and PDGF-BB on recovering and rescuing hypoxia-triggered damage to HCMs was basically consistent. Further, we added PDGF-BB^Ab^ into the COMP-treated group and surprised to find that the rescue capabilities of the compound induced hiPS-CMs were dramatically suppressed compared with that of the Hypoxia+COMP group (Fig. 7b-f).

### PDGF-BB secreted from the compound induced hiPS-CMs protected cardiomyocytes from apoptosis by upregulating PI3K and Akt hyperphosphorylation via activation of PI3K/Akt pathway

The levels of phosphorylation of PI3K and Akt involved in the regulation of cardiomyocytes repairing were compared among the Control, HYP, HYP + COMP, HYP + PDGF-BB and HYP + COMP + PDGF-BB^Ab^ groups (Fig. 7g). Treatments with COMP medium and PDGF-BB could counteract the negative effects of hypoxia and significantly increase the immunoblot reactivity of p-PI3K and p-Akt of hypoxia-injured HCMs alone, while the positive effects of phosphorylation of PI3K and Akt were reversed after the addition of PDGF-BB^Ab^ (Fig. 7h). Moreover, we repeated above experiments using another hiPSC lines BC and obtained similar results (Supplementary Fig. 9a–h). In addition, we combined compound induced hiPS-CMs with PDGF-BB^Ab^ for transplantation in vivo and found that UCG indexes including EF, FS, LVIDs and LVESV values were largely reversed (Fig. 7i), indicating that PDGF-BB secreted from compound induced hiPS-CMs played a key role in myocardial protection.

## Discussion

Even with the best current treatment, approximately 50% of heart failure patients die within 5 years of diagnosis. This figure is equivalent to or even far higher than the death rate of many common cancers.^17^ At present, there are two main methods of clinical treatment of heart failure: drugs and surgery. Heart transplantation is the only management option when advanced ischemic heart failure occurs and dilated cardiomyopathy is unresponsive to medical treatment, but the lack of heart donors and severe postoperative immune rejection have become urgent problems to be solved. Cell transplantation for myocardial injury is a promising approach of regenerative medicine, previous investigations have demonstrated that iPS differentiated cardiomyocytes transplanted into the scar tissue of the infarcted myocardium can survive and improve the myocardial perfusion and wall movement.^18–21^ Nevertheless, this treatment still has the disadvantage of arrhythmia complications. This problem may be related to the insufficient structural and functional maturity of cardiomyocytes differentiated from hiPS. Thus, we focused on optimizing the existing differentiation scheme to actively build more mature and stable cardiomyocytes in vitro, and further demonstrated the therapeutic potency of the optimized hiPS-CMs for heart failure.

It is very technically challenging to obtain large numbers of matured hiPS-CMs at high purity, even though a standard induction scheme by activating Wnt signaling shortly followed by inhibiting Wnt signaling and produced robust cardiomyocytes from hiPS had previously established.^22^ Thus, we tried to strengthen the standard induction scheme by adding our traditional Chinese medicine mixture, which was clinically effective in the treatment of patients with heart failure. Moreover, we were surprised to find that the differentiation rate and maturity of hiPS-CMs were significantly improved after adding Chinese medicine mixture. Then we screened the effective ingredients in Chinese medicine mixture to promote the functional maturation of hiPS-CMs and developed saponin^+^ compound with clear concentration and composition as our optimized compound induction scheme. An important aspect of this compound induction scheme is that the compound induced hiPS-CMs have more mature structure and stable electrophysiological functions, which are highly consistent with the late-stage of fetal heart development.

In this study, we verified that under the interference of activators and inhibitors of cardiomyocytes, the compound induced hiPS-CMs showed stronger sensitivity and stability than the standard induced hiPS-CMs in both the overall electrical signal transmission and the field potential response of single cells. Besides, we found that the main types of mitochondria in the compound induced hiPS-CMs were linear, branched and even reticular. We know that the mitochondria of mouse embryos are fragmented on day 9.5, and only on day 13.5 do the mitochondria develop branching and form a thin, filiform and interconnected network structure.^23^ It could be seen that the morphological maturation process of mitochondria in the compound induced hiPS-CMs is consistent with that of mitochondria during embryonic stages.

Conditions in the host blood circulation would influence the survival of transplanted cells, our optimally induced hiPS-CMs could maintain efficient differentiation in the fluid environment, providing possibilities for intravascular transplantation in vivo. Then, we transplanted the hiPS-CMs into immune-deficient heart failure mice and a slow heart rate was observed. It was possible that the transplanted hiPS-CMs reduced the oxygen consumption of the myocardium, making the heart muscle easier to be repaired. UCG confirmed that the compound induced hiPS-CMs were better than the standard induced hiPS-CMs in improving cardiac function and structural remodeling, and its therapeutic effect showed an increasing trend with time, while the therapeutic effect of the standard induced hiPS-CMs therapy was mainly confined to the early stage after transplantation, which might be related to the short time for standard induced hiPS-CMs to maintain aggregation to the heart. The same phenomenon was almost exactly reproduced in the micro-PET/CT examination with a result of promoting glucose uptake and improving energy metabolism in the injured myocardium, which was similar to the pharmacodynamic mechanism of the clinical drug trimetazidine.^24^

Previous study has shown that the main pathological features of myocardial failure are microvascular structural damage, myocardial apoptosis and fibrosis.^25^ In the present study, compound induced hiPS-CMs transplantation could significantly inhibit myocardial apoptosis and fibrosis and promote myocardial microvascular regeneration and structural reconstruction. Meanwhile, we found that GATA4, Nkx2.5 and HGF were significantly increased, while GATA4 and Nkx2.5 could inhibit myocardial apoptosis and fibrosis,^26,27^ and HGF could promote myocardial micro-angiogenesis,^28^ which explained the morphological changes of the myocardium after hiPS-CMs transplantation. Furthermore, we tested sensitive indicators such as BNP, CK, CK-MB, and LDH, which are commonly used in the clinical, and showed the effectiveness of the compound induced hiPS-CMs transplantation.

The mechanism of benefit deriving from cell-based therapy, including the contribution of new cardiomyocytes, angiogenesis, anti-inflammatory actions, anti-fibrotic actions, anti-apoptotic actions, and other effects.^29^ However, the mechanism through which these injected cells augment cardiac function has not been precisely determined. The recent publication showed that the mechanistic basis for cell therapy is not associated with the production of new cardiomyocytes, but due to an acute inflammatory-based wound-healing response that rejuvenates the infarcted area of the heart.^30^ This conclusion was also partially supported by our present results. We used real-time fluorescent tracking technology to dynamically track the transplanted hiPS-CMs in vivo and determined that the transplanted hiPS-CMs could circulate to the injured heart. Unfortunately, although hiPS-CMs homing to the heart was observed, no transplanted hiPS-CMs were imaged after hiPS-CMs transplantation. To verify the reliability of this result, we performed immunohistochemical staining for the human-specific cytoplasmic marker, SC121, in the mice heart at 1, 2, 4 and 8 weeks after hiPS-CMs transplantation, also received a negative result. Though hiPS-CMs were not integrated into the heart to play a direct repair role, they still showed a positive therapeutic effect, which confirmed the existence of other indirect modes of action, such as paracrine mechanisms.

In order to further explore the mechanism by which the compound induced hiPS-CMs outperformed the standard induced hiPS-CMs transplantation in myocardial repair, we used HCMs model of myocardial damage induced by hypoxia, which is a classic method to study heart disease in vitro. By using this model, we demonstrated that the conditioned medium from the compound induced hiPS-CMs could effectively recover and rescue hypoxia-injured HCMs, including improving cell viability, restricting cardiomyocytes apoptosis, reversing the expression of c-TNI protein in HCMs and decreasing the release of CK-MB. To understand the role of cytokines from the compound induced hiPS-CMs, we performed cytokine array analyses of the cell supernatants and three paracrine factors (PDGF-BB, lymphotactin and SCF) were found to be significantly more abundant in the compound induced hiPS-CMs compared to the standard induced hiPS-CMs. Among these three factors, lymphotactin is a chemokine that recruits T and NK cells.^31^ Recent study has also identified lymphotactin as a plasma indicator for LV dilation status,^32^ while the direct effect of lymphotactin on myocardial repair has not been reported in the literature. Another chemokine SCF has been proved that it can enhance chemotactic homing of myocardiocytes,^33^ which explains why the compound induced hiPS-CMs have more lasting ability to gather to the heart than the standard induced hiPS-CMs. However, there is no literature report on the repair of injured myocardium by SCF. Additionally, the previous study showed that PDGF-BB could protect various cell types from apoptotic cell death, and has direct or indirect effects on cardiomyocytes.^34^ Combined with our RNA-seq data and pathological results, which indicated that a key process for compound induced hiPS-CMs to protect myocardium is anti-apoptosis, we chose PDGF-BB as the entry point in our subsequent research of anti-apoptotic mechanism.

To verify whether PDGF-BB secreted from the compound induced hiPS-CMs could mediate the recovery of cardiomyocytes damage, PDGF-BB was added to the hypoxia-injured HCMs culture medium alone and showed almost the same ability of repairing cardiomyocytes as the conditioned medium from the compound induced hiPS-CMs. Then, PDGF-BB^Ab^ was added into the COMP-treated group and the multiple repair capabilities of the COMP medium were dramatically inhibited. Besides, PDGF-BB^Ab^ could reverse most of the protective effect of compound induced hiPS-CMs in vivo. These data supported an important role for PDGF-BB secreted from the compound induced hiPS-CMs in cardiomyocytes protection.

Through in vivo and in vitro models, we had more comprehensive evidence that the compound induced hiPS-CMs could effectively rescue the injured heart, possibly by regulating the activation of Akt activity. Upregulation of Akt has been reported to play a critical role in cell survival and angiogenesis,^35,36^ and protein Akt is activated in cardiomyocytes through the PI3K/Akt pathway.^37^ According to this, we investigated the regulatory relationship between PDGF-BB secreted from the compound induced hiPS-CMs and PI3K/Akt signal pathway under hypoxia conditions. We discovered that the COMP medium and PDGF-BB could counteract the negative effects of hypoxia and significantly increase the expression of p-PI3K and p-Akt, but the activation effects of phosphorylation of PI3K and Akt were restricted after the addition of PDGF-BB^Ab^. Thus, we can propose that the compound induced hiPS-CMs possess therapeutic potential by secreting PDGF-BB to upregulate PI3K and Akt hyperphosphorylation via regulating the PI3K/Akt pathway mechanistically. In addition, our study suggests that a well-defined compound mix may be a promising stimulator on a specific cell lineage differentiation from stem cells for a broad spectrum of usages scientifically and clinically.

However, the present study had some limitations. The animal models we used were isoproterenol-induced heart failure, which were similar to clinical acute and subacute heart failure caused by ischemia. Although our cell transplantation method is effective in this mice model, it is still unknown whether it is effective in humans and other animals or in large area myocardial infarction models. Further investigation will be carried out in primates and develop various PDGF-BB combination therapies for heart failure will become our key work in the future.

In conclusion, our highly and optimally differentiated hiPS-CMs system can serve as a promising approach for the treatment of heart failure clinically. Our study showed that the compound induced hiPS-CMs could improve cardiac function in the context of heart failure, primarily through paracrine cytokine effects. This newly developed hiPS-CMs system may provide a basis for clinical cell transplantation, which has enormous therapeutic potential for promoting the repair of the damaged myocardium. Mechanistically, the structural and functional recovery, as well as the repairing of injured cardiomyocytes elicited by transplanted hiPS-CMs induced by saponin^+^ compound, were mainly mediated by PDGF-BB secreted from the compound induced hiPS-CMs through the activation of PI3K/Akt signaling pathway. On top of this, such system can be used to study the etiology of unexplained heart disease, offering an ideal in vitro experimental model for drug metabolism, toxicity detection and new drug development. Collectively, we suggest that PDGF-BB used in isolation or in combination with the compound induced hiPS-CMs may be a promising method to prevent and treat heart failure, which is of particular interest for future research.

## Materials and methods

### Ethics approval and statements

Animal experiments were performed in accordance with the framework of the Institutional Animal Care and Use Committee of Beijing University of Chinese Medicine.

Immunofluorescence staining, karyotype analysis, LC–IT-TOF-MS measurements, microfluidics experiments, extracellular electrograms recording and calcium transients tests, flow cytometry, fluorescence imaging

Detailed descriptions for experimental procedures are provided in the supplementary materials online, Methods.

### Cell culture

Human-induced PSC (hiPSC) lines UC were obtained from Beijing Saibei Biotechnology Company Ltd., and the pluripotency and karyotype of the hiPSC were confirmed (Supplementary Fig. 1). The passage number of the hiPSC ranged from 20 to 30, and they were cultured on Matrigel (BD Biosciences)-coated plates and maintained in mTeSR^TM^ medium (STEMCELL Technologies).

P19 mouse embryonal carcinoma cells were obtained from Wuhan Punosai Life Technology Ltd., and these cells were cultured in α-MEM medium (10% FBS, 1% penicillin and streptomycin) in T25 flasks. The cultures were split 1:20–1:100 at 80–90% of confluence using 0.02% EDTA solution in PBS (2 min incubation at 37 °C, 5% CO_2_).

### Chinese medicine mixture preparation

The Chinese medicine mixture (abbreviated as “Mix”) is a new traditional Chinese medicine prescription developed for cardiomyocyte differentiation based on our previous research experience.^15^ It is mainly comprised of sea dragon, pilose antler, sea horse, ginseng, astragalus, epimedium, dried deer placenta, salvia miltiorrhiza, gynostorium pentaphyllum, and saussurea involucrate.

### Saponin^+^ compound preparation

The saponin^+^ compound (abbreviated as “compound”) is our patent pending product developed from the Chinese medicine mixture. It is mainly comprised of icariin, salvianolic acid B, sphingosine-1-phosphate, astragaloside, ginsenoside rg1, PLGF-2, new isomer of scropolioside D with a well-defined molecular profile (Fig. 1c). Addition of the saponin^+^ compound at the optimal dose was used to promote differentiation and maturation of cardiomyocytes in our system.

### Optimized cardiomyocytes differentiation

Before the initiation of cardiomyocytes differentiation, hiPSCs were dissociated into clumps using Dispase (Sigma-Aldrich) and were plated onto Matrigel Basement Membrane Matrix Growth Factor Reduced (BD Biosciences). When the cells attained a confluence of 90–100% (depending on the cell line), mTeSR was replaced with CDM medium containing 10 μM CHIR99021 (Sigma-Aldrich) for 24 h. The medium was then replaced with CDM medium containing 5 ng/mL bFGF (Peprotech) for 1 day, followed by CDM medium with or without the saponin^+^ compound, then cultured for another day. Next, the cultures were changed to half of the CDM medium containing 5 μM IWP2 (Sigma-Aldrich) with or without the saponin^+^ compound for 2 days. Finally, the differentiated cells were cultured in RPMI1640/3% KnockOut™ Serum Replacement (Gibco) medium with or without the saponin^+^ compound for further growth. The medium was changed every 48 h. On Days 10, 11, 13, and 15, the medium was replaced with glucose-free DMEM (Invitrogen) containing 4 mmol/L L-lactic acid (Sigma) to metabolically select and purify cardiomyocytes (hiPS-CMs). 15 days after starting the differentiation, the optimally induced hiPS-CMs exhibited spontaneous contraction were used for transplantation into mice.

### Construction of recombinant luciferase reporter gene plasmid psiCheck-2-Myh6

The 3’ UTR of Myh6 was cloned at the Xho1 site of the psiCheck2 plasmid (Supplementary Fig. 2). The 5’ UTR of Myh6 was cloned at Nhe1 site. The double clones including both 5’ UTR and 3’ UTR were cloned in their respective sites (upstream and downstream of RLuc). Recombinant plasmids were fully sequenced in both directions before use.

### Luciferase assay

Transfections with expression vectors (0.5 μg per transfection) were carried out in P19 cells at 70% confluence using Lipofectamine-3000 (Life Technologies) according to the supplier’s instructions. The medium was replaced after 6 h of transfection with fresh low-serum differentiation media and cultured for 10 days before determination of luciferase activities using the Dual-Luciferase system. The Firefly and Renilla luciferase activities were measured using the Dual-Glo Luciferase assay system (Promega) according to the manufacturer’s instructions. The ratio of Firefly luciferase activity to Renilla luciferase activity was used to normalize any differences in transfection efficiency among samples. All luciferase analyses were performed in three independent experiments.

### Animal modeling and hiPS-CMs transplantation assay

Four- to six-week-old male BALB/cA-nu mice (immunodeficient nude mice), weight between 16–18 g (purchased from Beijing Huafukang Biotechnology Co., Ltd.) were used as recipients. Heart failure model mice were induced by intraperitoneal injection of isoproterenol (ISO) at a dose of 20 mg/kg body weight on day 1, 10 mg/kg body weight on day 2, and subcutaneous injection of ISO 5 mg/kg on days 3–14. After successful establishment of the heart failure model, 2 million (2 × 10^6^) purified hiPS-CMs were washed and suspended in 250 μL PBS and then transplanted by tail intravenous injection. At 4 and 8 weeks after transplantation, the mice were humanely euthanized and subjected to further experimentation. For tracking the transplanted cells, the hiPS-CMs were stained with CM-DiL fluorescent dyes (Supplementary Fig. 4a) and identified with cardiac-specific marker (Supplementary Fig. 4b) before transplantation.

### Fluorescence imaging of hiPS-CMs transplantation in vivo

Fluorescence imaging was acquired after intravenous injection of hiPS-CMs using the VISQUE Invivo Smart-LF apparatus (Vieworks. Inc, Korea) with the 553 nm excitation filter and the 570 nm emission filter. Fluorescence imaging was continuously observed for the following 20 days, and representative photographs were acquired.

### Electrocardiography measurements

Mice were anesthetized using a mixture of isoflourane and oxygen. Anesthetized mice were secured in a supine position on a regulated heat pad while three-lead electrocardiography was performed (PowerLab/4SP System and ML136 Animal Bio Amp module, ADInstruments, Spechbach, Germany). A recording time of 2 min was considered sufficient to provide a representative view of heart function. The data recorded were submitted to a 50-Hz notch filter with an automatic setting determined by the software.

### Echocardiographic analysis

Anesthetized animals were placed on a mouse bed in a shallow left lateral decubitus position. Transthoracic echocardiography was performed using a pediatric broad band 6–15 MHz linear array ultrasound transducer (Agilent Sonas 5500; Agilent Technologies, Palo Alto, USA). The ultrasound beam depth was set at 2 cm and the frame rate at 150 frames/s. The two dimensional parasternal short-axis views were obtained at the level of the LV papillary muscles. All measurements were averaged on 3 consecutive cardiac cycles and analyzed by a single observer who was blinded to the status of the animals.

### [^18^F]-DPA-714-PET/CT imaging and analysis

The mice were anesthetized and injected with 10MBq/0.1 ± 0.2 mL of ^18^F-DPA-714 via the tail vein. Thirty min after the injection, the mice were maintained under isoflurane anesthesia (2% in 100% oxygen) on a heating pad and imaged with PET/CT (Inveon small animal scanner, Siemens, Berlin, Germany). Static whole-body PET scans were performed during 10 min in 2 frames. CT scan (40 kV, 250 mA) was obtained during 11.5 min in two frames. The images were reconstructed using Inveon Research Workplace4.2 (Matrix size; 256 × 256) by the software module of the scanner. The data were reconstructed over a 175 × 175 × 118 matrix with 0.79-mm slice thickness. Fused images were converted to DICOM format and analyzed with OsiriX shareware (Geneva, Switzerland) by manually selecting regions of interests (ROIs) as the anterior wall and the septal wall of the LV for calculation of the maximum standard uptake values (SUVmax), which were calculated as follows: SUV = radioactivity in ROI (Bq/cm^3^)/injected dose (Bq)/body weight (g). To minimize the effects of injection errors, the uptake ratio was used to compare SUVmax among the different models and defined as follows: Uptake ratio = (SUVmax in the anterior LV wall)/(SUVmax in the septal LV wall). ^18^F-DPA-714-PET/CT imaging was performed in the fourth and eighth week after the hiPS-CMs transplantation.

Transmission electron microscopy, ELISA assay, RT-PCR tests, cell viability assay, cytokine antibody array, western blotting, and RNA sequencing

Detailed descriptions for experimental procedures are provided in the supplementary materials online, Methods.

### Statistical analysis

All statistical analyses were performed using SPSS 21.0. For comparison between two mean values, a two-sided Student’s *t-*test was used to calculate statistical significance. Two-way ANOVA tests were performed followed by Bonferroni *t*-tests for all pairwise multiple comparison procedures. A *P*-value < 0.05 was considered to be significant.

## Supplementary information

Supplementary materials

Supplementary figure 3

Movie S1. Spontaneous pulsation properties of standard induced hiPS-CMs on day 15 of differentiation

Movie S2. Spontaneous pulsation properties of compound optimally induced hiPS-CMs on day 15 of differentiation

## Data Availability

The datasets used and/or analyzed during the current study are available from the corresponding authors on reasonable request.
