# Supplementary material for: Cardiomyocytes induced from hiPSCs by well-defined compounds have therapeutic potential in heart failure by secreting PDGF-BB
Source: Signal Transduct Target Ther. 2022 Jul 29;7:253. doi: 10.1038/s41392-022-01045-4 (PMC9334380; doi:10.1038/s41392-022-01045-4)
Supplement: Supplementary file 1 — Supplementary materials [file 41392_2022_1045_MOESM1_ESM.docx]

Supplementary Materials for

Cardiomyocytes induced from hiPSCs by well-defined compounds have therapeutic potential in heart failure by secreting PDGF-BB

Hongmei Li (MD)^a, c^, Fenfang Wu (PhD)^b^, Guangrui Huang (PhD)^a^, Di Wu (PhD)^d^, Ting Wang (PhD)^a^, Xiashuang Wang (PhD)^d^, Kai Wang (MD)^c^, Yuyin Feng (MS)^a^, Anlong Xu (PhD)^a, d, *^

Correspondence to: xuanlong@bucm.edu.cn

**This PDF file includes:**

Materials and Methods

Figures. S1 to S9

Tables S1 to S3

Captions for Movies S1 to S2

Materials and Methods

**Pluripotency and karyotype identification of hiPSCs.**

**Immunofluorescence and karyotype analysis**

Immunofluorescence was performed against NANOG, OCT4, SOX2 and SSEA-4. Slides were blocked and antibodies diluted using 1× casein-10%-normal serum-PBST (Vector Laboratories, Burlingame, CA). Primary antibodies were incubated overnight at 4°C. Secondary antibodies were incubated for 1h at room temperature. Slides were stained with DAPI during washing and coverslips mounted with VECTASHIELD HardSet mounting medium (Vector Laboratories). Karyotype analysis of the iPS cell chromosomes was carried out using a standard G-band technique (300-400 band level).

**LC–IT-TOF-MS measurements**

A Shimadzu LC–IT-TOF-MS system (Kyoto, Japan) consisting of two LC-20ADXR pumps, a DGU-20A3R degasser, a SIL-20ACXR auto-sampler, a CTO-20AC column oven, a CBM-20 controller, a SPD-M20A DAD module, and a hybrid ion trap-time of flight-mass spectrometer (IT-TOF-MS). Chromatographic separations were conducted on a Waters Acquity UPLC HSS T3 column (2.1×100mm, 1.8μm, Milford, MA, USA), and the connection from LC to the mass spectrometer was achieved via an electrospray ionization (ESI) interface. The mobile phase composed of 0.1% formic acid (A) and ACN (B) was delivered in gradient as following program: 0-15min, 30%-75%B; 15-20min, 75%-100% B; 20.1-25min, 30% B, with the flow rate of 0.25mL/min. The injection volume was set at 5.0μL and the column oven was maintained at 40°C. The column outlet was successively connected to DAD module and IT-TOF-MS. The key parameter settings of IT-TOF-MS followed the descriptions in the literature. LC-MS solution software (Version 3, Shimadzu) took the load of data processing and a mass tolerance of ±10 ppm was defined for molecular formula calculation.

**Comparison of the stability of differentiated hiPS-CMs between the standard and compound induction schemes at micro and macroscale**

**Using a microfluidic device to record continuous differentiation imaging with media flow**

For the microfluidics experiments, hiPS cells growing in the exponential phase were injected into a CellASIC microfluidics chamber (ref. M04S-03, Merck-Millipore, Darmstadt, Germany), using the Microfluidic Perfusion Platform (Supplementary Fig. 3a) driven with the interface software ONIX-FG-SW (Merck-Millipore). The cells were trapped and maintained in a uniform plane (Supplementary Fig. 3b). Normal growth conditions were reproduced by adjusting the ambient temperature to 37°C with a thermostated chamber, and by flowing cells with the indicated different inductive differentiation medium at 1 psi. The microfluidics device was coupled to a DMI6000 (Leica, Buffalo Grove, IL) microscope, equipped with a QuantEM cooled EMCCD camera (Photometrics, Tucson, AZ) and a spinning-disk confocal system CSU22 (Yokogawa, Tokyo, Japan). Image resolution was 1 pixel = 149nm and images were acquired with MetaMorph 7 software (Molecular Devices, Sunnyvale, CA).

**Extracellular electrograms recording**

Unipolar electrograms were recorded from hiPS-CMs plated on MEAs. By calculating the LAT at each electrode, we generated an activation map, demonstrating that the activation propagated from the upper right corner to the bottom left corner. Conduction velocity, propagation pattern, depolarization, repolarization, beat timing and arrhythmias were tested.

**Intracellular Ca^2+^ measurement**

To record calcium transients, the hiPS-CMs were incubated with 2μM Fluo-4 AM (Life Technologies) in HEPES buffered saline solution (140mM NaCl, 2.8mM KCl, 2mM CaCl_2_, 2mM MgCl_2_, 10mM glucose, and 10mM HEPES, pH 7.4) at 37°C for 30min to allow for de-esterification of the dye that had penetrated the cell membrane. After removing the excess dye in the buffer, the cells were washed 3 times with PBS for 10min, and the spontaneous Ca^2+^ transients were recorded at 37°C using an Olympus IX81 motorized inverted fluorescence microscope and a time-lapse recording system (Xcellence).

**More mature structure and function of mitochondria in the compound induced hiPS-CMs than that in the standard induced hiPS-CMs**

**Detection of mitochondrial structure and function in hiPS-CMs**

*Rhod-2 fluorescence digital imaging.* For the qualitative measurement of [Ca^2+^], hiPS-CMs were plated three or more days before the experiment on Fluorodish (Ibidi) with an initial plating density of 6,500 cells/cm^2^. Then, the cells were loaded with 1μM rhod-2/AM (Abcam) in a 37°C incubator for 45min and washed thoroughly. Next, the cells were incubated in a 37°C incubator for 30min on a medium containing 5μg/ml Hoechst 33342 (Invitrogen) and 100nM MitoTracker Green/FM (Thermo). The data were collected under 405, 488 and 555nm excitation waves under confocal microscopy. The fluorescence intensity of Rhod-2 was measured by ZEN software and compared with the average fluorescence intensity of MTG-labeled mitochondria. High resolution imaging enabled identification of individual mitochondria for analysis.

*TMRE fluorescence digital imaging*. For analyses of changes in mitochondrial membrane potential (Δψm), cells were loaded with 100nM of tetramethylrhodamine ethyl ester (TMRE), 100nM mitochondrial green fluorescent probe (MTG) and 5μg/ml Hoechst 33342, and incubated in an incubator for 30min. TMRE fluorescence was excited at 546nm and emitted fluorescence was collected through a 590-nm-longpass barrier filter.

*Analysis of cytosolic cytochrome c (Cyto C) release.* After hiPS-CMs were maintained in RPMI1640/3% KnockOut™ Serum Replacement medium without or with the saponin^+^ compound, the release of cyto C from the mitochondria to the cytoplasm was investigated by immunofluorescence.

**Detection of hiPS-CMs differentiation rate by flow cytometry**

A total of 1×10^6^ cells were incubated with TrypLE (Gibco) at 37°C until the cells began to dissociate. Cells were centrifuged and resuspended in 2% PFA at 4°C for 20min and washed with PBS, then permeabilized in PBS containing 0.1% Triton X-100 and 5% FCS for 30min. Cells were then incubated for 2h in the dark with c-TNI antibody (BD; 1:20) on ice. No antibody was added for the negative control. Finally, the cells were washed with PBS containing 0.1% Tween-20 and measured using a FACS LSR Fortessa flow cytometer (BD Biosciences, FACS Aria II).

**Comparison of the differences and similarities in maturity among standard/compound-induced hiPS-CMs and fetal/adult hearts**

**Data source and data preprocessing**

RNA-seq datasets GSE106688 (fetal heart) and GSE116250 (adult heart) obtained from the Gene Expression Omnibus (GEO, http://www.ncbi.nlm.nih.gov/geo/) database were sequenced on the platform of GPL18573 and GPL16791, respectively. The raw data of GSE106688 (fetal heart), GSE116250 (adult heart) and our RNA-seq data (STD and COMP) were normalized for analysis. The shared genes of GSE106688 (fetal heart), GSE116250 (adult heart) and our RNA-seq data (STD and COMP) were obtained, then PCA (Principal Component Analysis) of these shared genes was completed using the Wekemo Bioincloud (website: https:// www.bioincloud.tech). Further, Venny 2.1.0 (website: https://bioinfogp.cnb.csic.es/tools/venny/index.html) was used to perform intersectional gene mapping. Heatmap of "STD vs. fetal" and "COMP vs. fetal" for shared genes related to early or late-stage of heart development were from Wekemo Bioincloud. Stacked bar plots (GraphPad Prism 8) was performed on early and late-stage genes of heart development to determine the maturity of STD and COMP in which stage of fetal myocardial development.

**Safety and efficacy of the compound induced hiPS-CMs transplantation confirmed by *in vivo* assay**

**Histology and immunofluorescence**

H&E staining were performed by standard methods using 5µm paraffin sections. Fibrosis and apoptosis were measured in hearts transplanted with hiPS-CMs by using Masson's Trichrome and TUNEL assays. Immunofluorescence was performed on 5μm frozen sections using antibodies against α-SMA and c-TNI. Sections were blocked and antibodies diluted using 1× casein-10%-normal serum-PBST (Vector Laboratories, Burlingame, CA). Primary antibodies were incubated overnight at 4°C. Secondary antibodies were incubated for 1h at room temperature. Slides were stained with DAPI during washing and coverslips mounted with VECTASHIELD HardSet mounting medium (Vector Laboratories). All antibodies are listed in Table S1.

**Transmission electron microscopy**

Ultrathin sections of heart tissue were fixed in 4.5% glutaraldehyde in 0.1M phosphate buffer pH 7.2 (PB). After washing with 0.1M PB, specimens were subsequently fixed for 1h with 1% osmium tetroxide in PB and washed with water. Specimens were then dehydrated in ascending concentrations of ethanol including en-bloc contrasting using 2% uranylacetate in 70% ethanol for 1h. Subsequently, they were used for the preparation of ultrathin sections that were post-stained with 2% uranylacetate and 0.2% lead citrate. The sections were observed using a LIBRA 120 transmission electron microscope (Carl Zeiss, Germany).

**Detection of paracrine factor by ELISA assay**

Cardiac paracrine factors VEGF, HGF, TGF-β and BNP quantities in mouse serum were determined using the mouse Quantikine ELISA Kit (Dogesce System) according to the manufacturer's instructions. Quantities were estimated based on a standard curve generated with cardiac paracrine factor.

**RNA isolation and RNA-seq analysis**

Total RNA was extracted from the hearts of mice and qualified using an Agilent 2100 bioanalyzer with an RNA Nano 6000 Assay Kit (Agilent Technologies, CA, USA). For library preparation and sequencing, library construction was performed using a TruSeq stranded mRNA library prep kit LT (Illumina, San Diego, CA, USA) according to the manufacturer’s instructions. Library quality and size distribution were determined with the Agilent Bioanalyzer DNA-1000 Kit (Agilent Technologies) and the average sizes of the libraries were approximately 260bp, as recommended by Illumina. To pool equal amounts of libraries with different adaptors indexes, quantitative PCR was performed using Bio-RAD CFX 96 KIT IQ SYBR GRN. All sequencing was performed on a NextSeq 500 system (Illumina) using a NextSeq500 mid-output v2 paired-end sequencing kit, 150 cycles (Illumina). RNA was isolated from the hearts of three mice per group with three biologic replicates for RNA-Seq analysis.

**Gene Oncology (GO) and KEGG function enrichment analysis**

GO and KEGG function enrichment analyses were carried out through the Annotation, Visualization and Integrated Discovery (DAVID) bioinformatics resources 6.8 (<https://david.ncifcrf.gov/>). We set｜Log2FC｜≥ 1 and *P* adjust < 0.05 as the threshold for differentially expressed gene (DEGs) screening. Enrichment analysis was performed after uploading DEGs and selecting the species as *mus musculus*. *P* value < 0.05 was considered strongly enriched in the annotation categories and was accepted for this analysis.

**mRNA analysis by Real-Time PCR**

mRNA (1μg) was reverse transcribed into cDNA via Oligo (dT) with SuperScript III Reverse Transcriptase (Invitrogen). PCR was performed using a Go Taq polymerase kit (Promega) with the following conditions: 95 °C for 2min; followed by 34 cycles of 94°C for 30s, 60°C for 30s, and 72°C for 45s; followed by a single cycle of 72°C for 5min. Relative quantification was performed against a standard curve and the values were normalized against the housekeeping gene, glyceraldehyde 3-phosphate dehydrogenase (GAPDH). The primers used are listed in Table S2.

**PDGF-BB secreted from the compound induced hiPS-CMs rescued the cell model from hypoxia-induced human cardiac myocytes injury and played a key role in the recovery of cardiac function *in vivo*.**

**Human cardiac myocytes culture and drug treatment**

Human cardiac myocytes (HCMs, C-12811, PromoCell, Heidelbery, Germany) were isolated from normal human ventricle tissue of the adult heart and used at passage 2-8. HCMs were cultured in a myocyte growth medium (C-22070, PromoCell) containing 5 µg/mL insulin, 5% FCS, 2 ng/mL fibroblast growth factor (FGF), and 0.5 ng/mL epidermal growth factor (EGF). During this period, culture medium was replaced every week. For cell treatment, hypoxia-damaged HCMs were replaced with COMP medium or 10ng/mL PDGF-BB (Proteintech, HZ1308), while Con and HYP groups were replaced with myocyte growth medium. To neutralize the function of PDGF-BB in COMP medium, 0.5 µg/mL PDGF-BB antibody (PDGF-BB ^Ab^) (R&D, AF-220-SP) was used in the experiment.

**Establishment of hypoxia HCMs model**

The cell model of hypoxia-injured human cardiac myocytes (HCMs) was established by 12 hours of continuous hypoxia under the culture conditions of 95% N_2_ and 5% CO_2_.

**Cell viability assay**

The cell viability of hypoxia-injured HCMs was detected by using cell counting kit-8 (CCK-8, AbMole BioScience, Houston, TX, USA). Cells were seeded in 96-well plates with a density of 2×10^4^ cells/well. Approximately 20μl CCK-8 reagent was added to each well, incubated at 37°C for 10min, and then placed on a shaker for five minutes. The absorbance value (OD) at 450nm was measured by using ELISA reader. Each assay was performed in triplicate and repeated three times. Cell vitality was calculated based on the following formula: cell vitality = the OD value of the experimental group/that of the control group * 100%.

**Human Cytokine Antibody Array**

To determine the secretory profile of the conditioned medium generated from the hiPS-CMs, the supernatant acquired from standard and compound induced hiPS-CMs culture were stored at −80°C, and were thawed before use in accordance with manufacturer’s guidelines provided by RayBio C-Series Human Cytokine Antibody Array (AAH-CYT-G2000-4; Raybiotech, Norcross, GA, USA). Experimental steps and analyses were conducted according to the manufacturer’s instructions. ChemiDoc^TM^ XRS (Bio-Rad, California, USA) was used for detection and Image Lab software (Bio-Rad, CA, USA) was used for quantification.

**Western blot analysis**

Protein samples were prepared by homogenizing whole hearts extracted from the mice. Each 50mg of heart tissue sample was lysed in 500μl of protein RIPA lysis buffer (Santa Cruz Biotechnology, CA, USA) and centrifuged. Tissue lysates were equalized and separated by electrophoresis using a 10% polyacrylamide gel, then transferred for 1h to nitrocellulose membranes (Bio-Rad). The membranes were blocked for 1h in nonfat milk (5%). After blocking, the membranes were probed with primary antibodies against c-TNI. Primary antibody binding was detected by HRP-conjugated secondary antibodies and enhanced chemiluminescence reagents. All antibodies are listed in Table S3.

**Animal treatment**

Heart failure model mice were induced by intraperitoneal injection of isoproterenol (ISO). After successful establishment of the heart failure model, 2 million (2×10^6^) purified hiPS-CMs were washed and suspended in 250μL PBS with or without 0.5 µg/mL PDGF-BB ^Ab^ and then transplanted by tail intravenous injection. At 4 and 8 weeks after transplantation, the mice were received routine echocardiography examination.

**
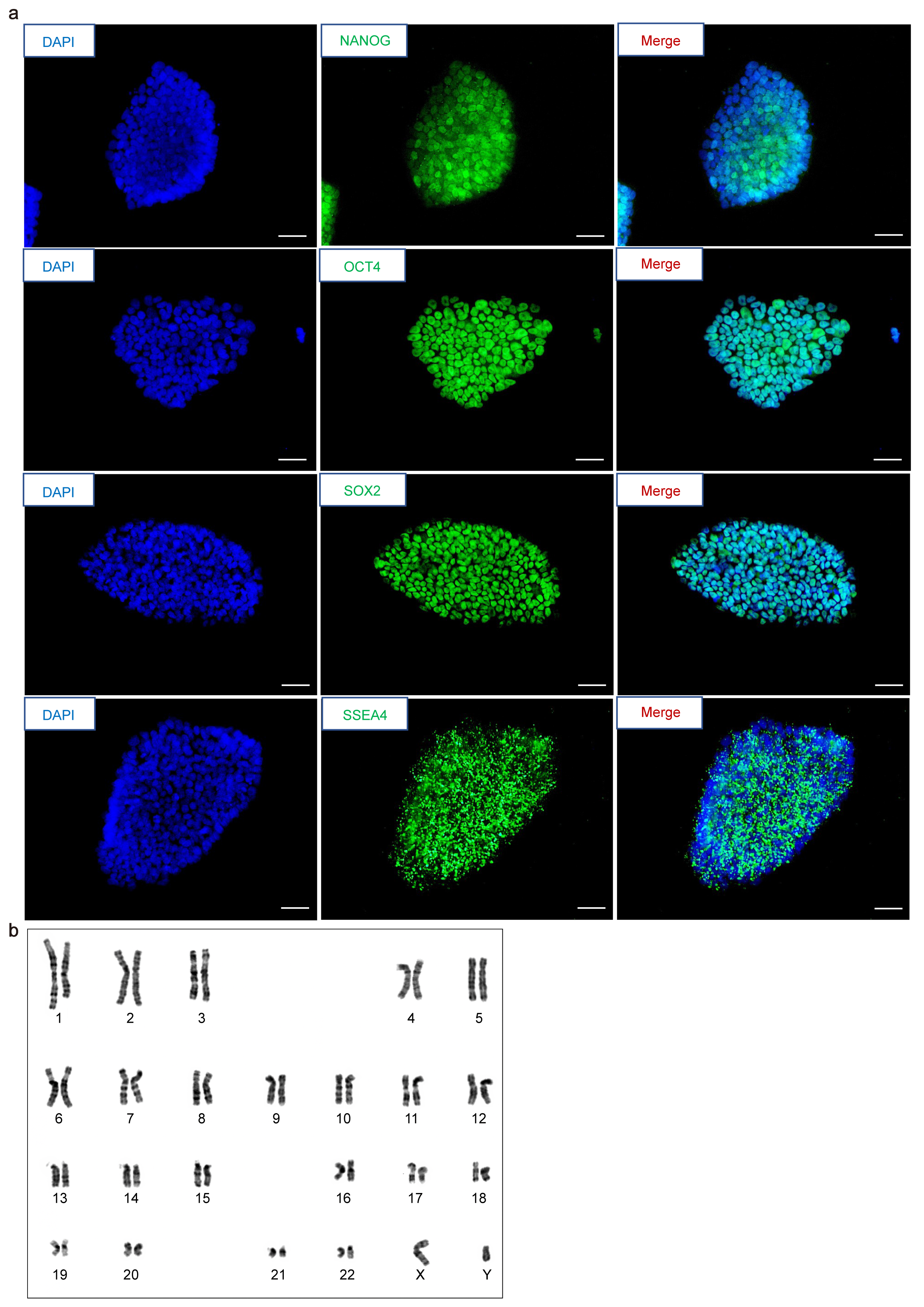
**

**Figure. S1.**

Pluripotency and karyotype identification of hiPSCs.

a, Immunofluorescent analysis of pluripotency markers (NANOG, OCT4, SOX2, SSEA4).

b, Normal karyotype of hiPSC was evaluated by G–banding analysis. All scale bars = 50 μm. hiPSCs, human induced pluripotent stem cells.


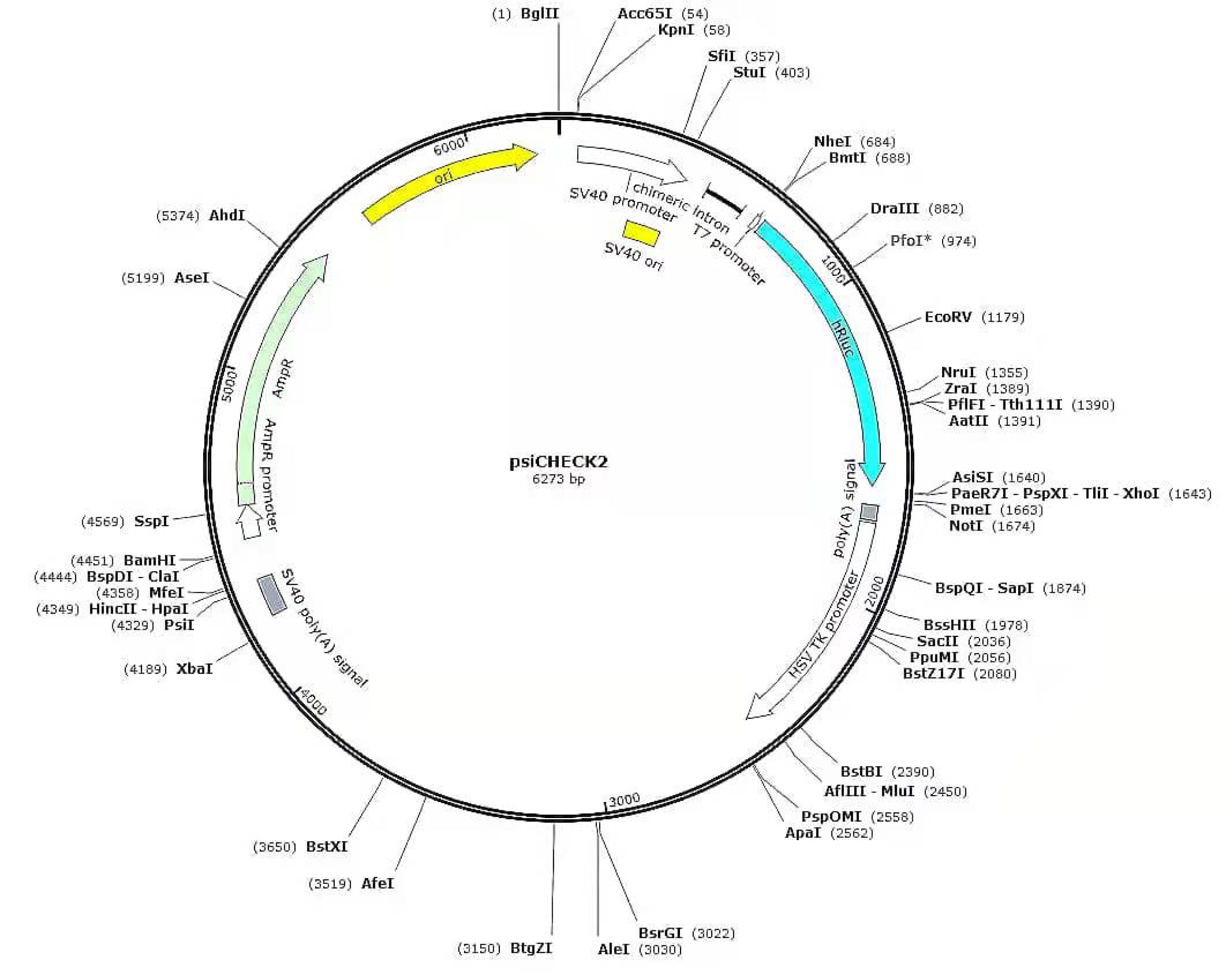


Figure. S2.

Vector structure map of psiCHECK-2.


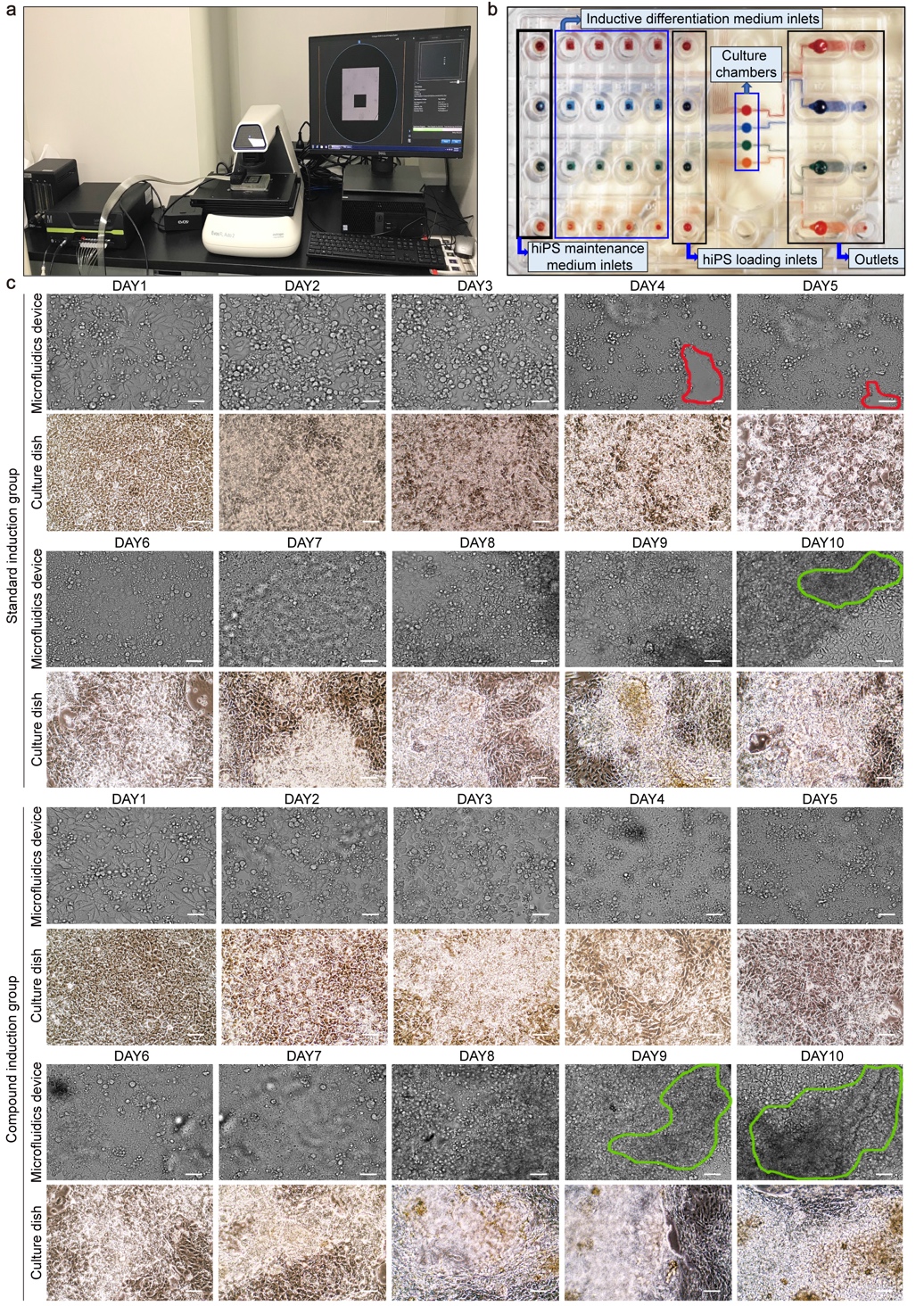


**Figure. S3.**

CellAISC^®^ ONIX2 microfluidic chip perfusion system simulated the microenvironment following hiPS-CMs venous implantation *in vitro*.

a, Images of the microfluidic cell-culture system.

b, Rubber/silicone tubing is used to apply pressure to the well-plate inlets.

c, Comparison of morphological changes in the differentiation of hiPSCs to cardiomyocytes under the standard and saponin^+^ compound induced protocols over 10 days in culture (the area of cell loss is marked in red, the area of cell pulsation is marked in green). Scale bars = 50 μm. hiPSCs, human induced pluripotent stem cells.


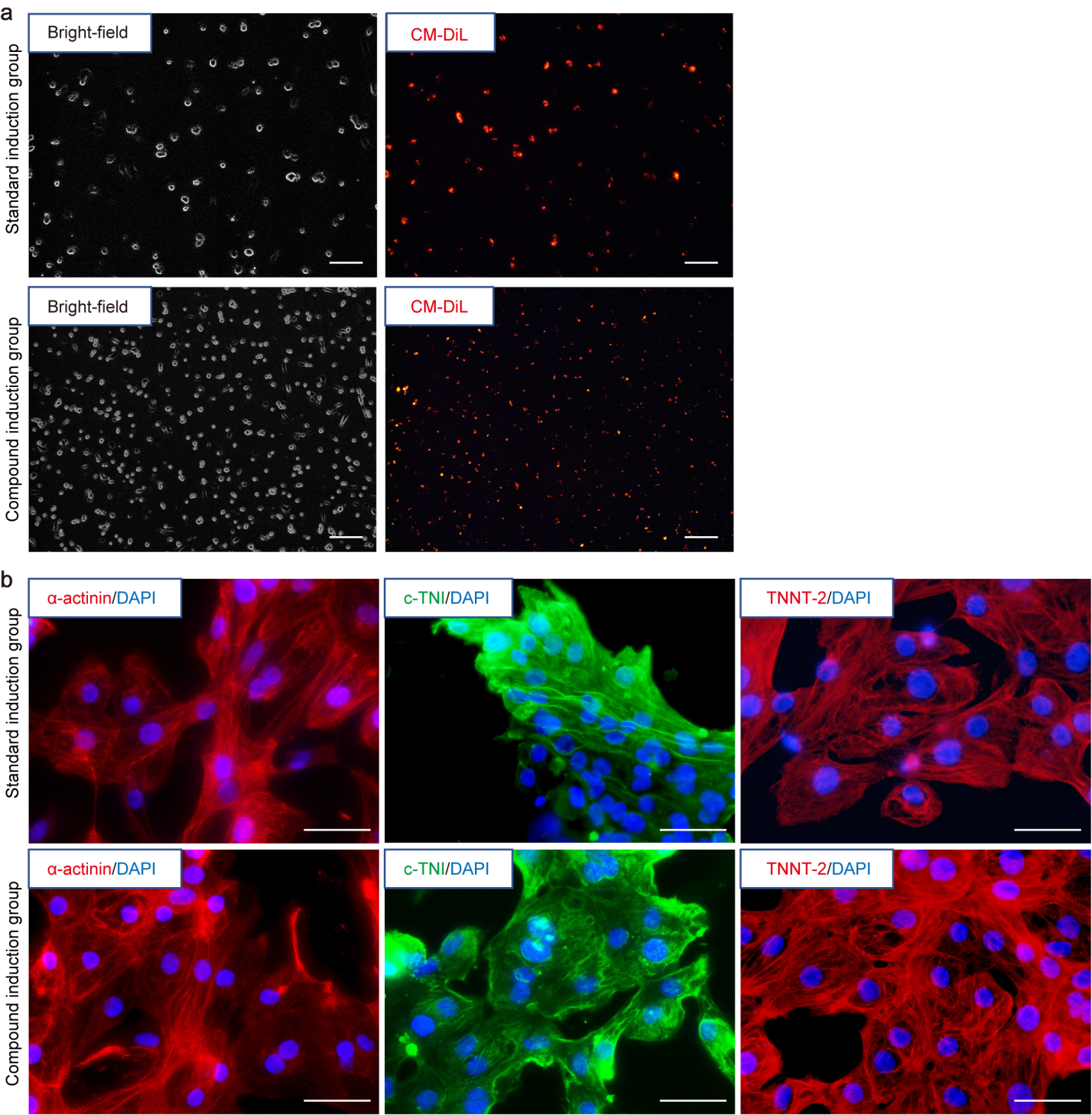


**Figure. S4.**

Identification and labeling of hiPS-CMs.

a, Fluorescence micrograph of CM-DiL labeled suspended hiPS-CMs. More than 90% of cells exhibited red fluorescence.

b, hiPS-CMs express the cardiac-specific markers including c-TNI, α-actinin and TNNT-2. All scale bars=50 μm. hiPS-CMs, cardiomyocytes derived from human induced pluripotent stem cells.

**
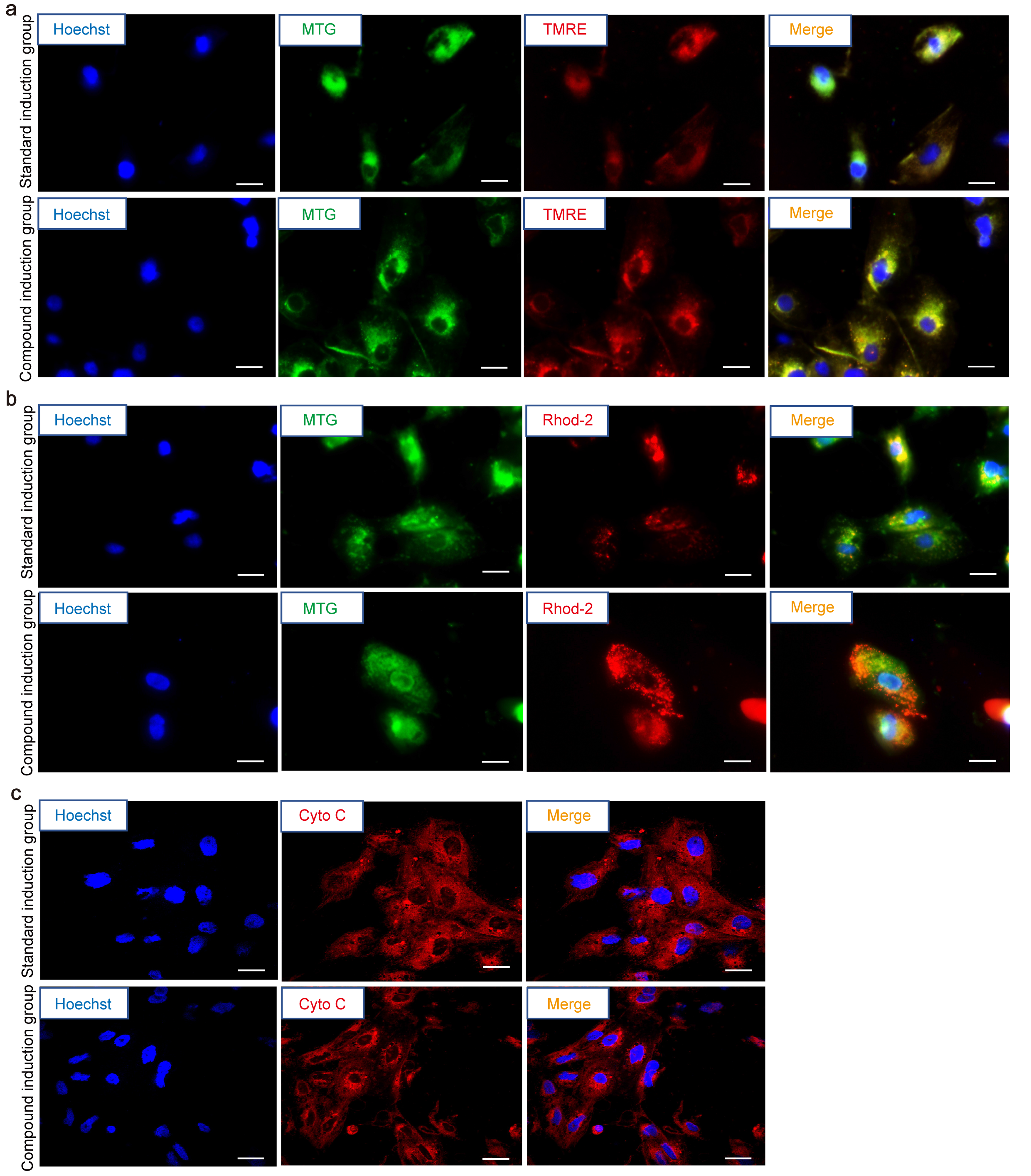
**

**Figure. S5.**

Representative photomicrographs of mitochondria related indexes of the compound and standard induced hiPS-CMs.

a, The mitochondria were labeled with tetramethylrhodamine methyl ester (TMRE) to detect the mitochondrial membrane potential and analyzed with a fluorescence microscope.

b, Detection of mitochondrial calcium level in hiPS-CMs by Rhode-2 fluorescence staining.

c, Immunofluorescence staining for cyto C in hiPS-CMs differentiated under the standard and saponin^+^ compound induction protocols. Both groups of cyto C were retained in the mitochondria. All scale bars = 50 μm.


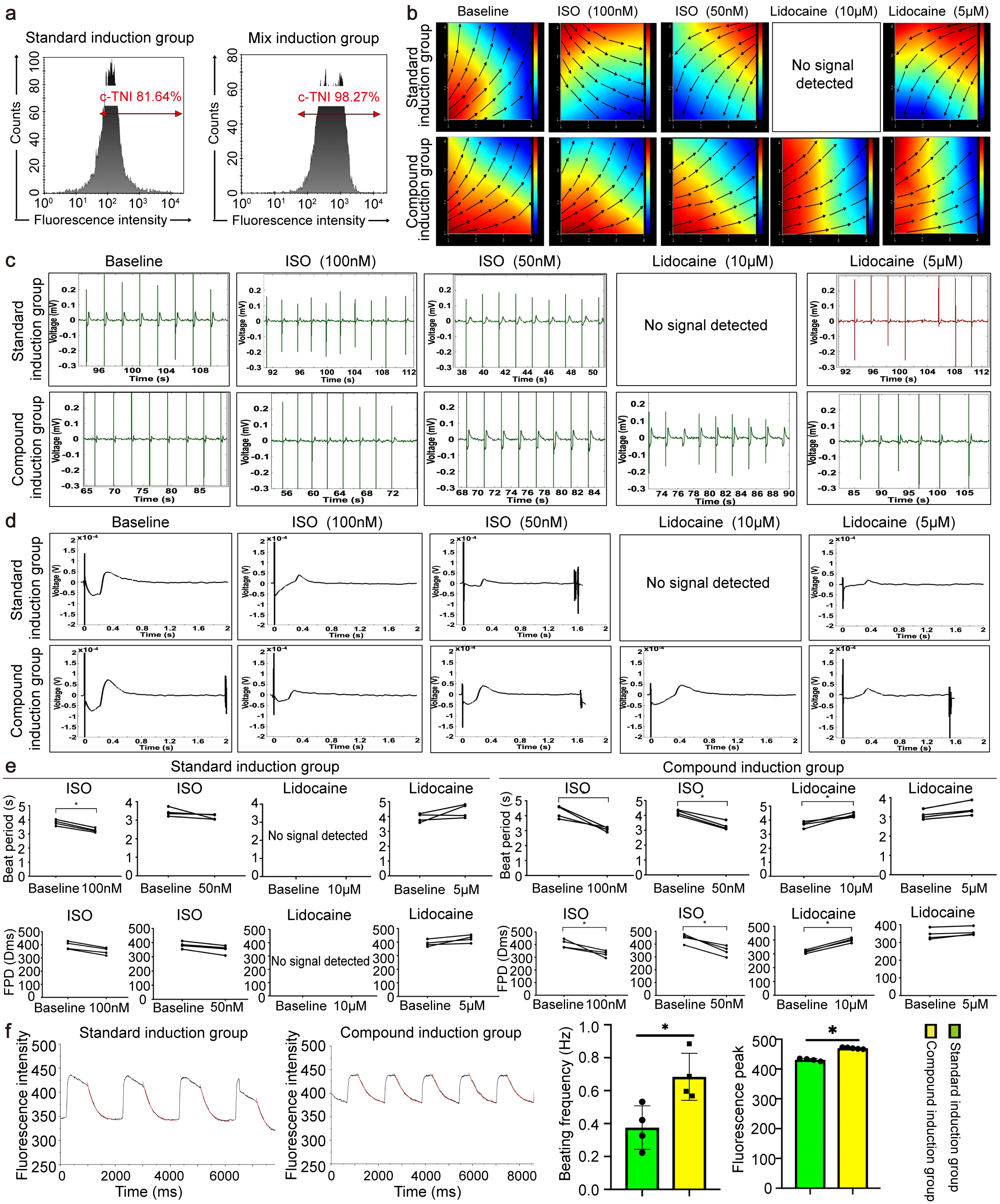


**Figure. S6.**

Comparison of extracellular electrograms and intracellular Ca^2+^ measurement of the standard and compound induced hiPS-CMs from hiPSC lines BC (reprogrammed from blood cells).

a, Flow cytometry was used to detect the positive rate of hiPS-CMs optimally induced by Chinese medicine mixture. Representative histogram of hiPS-CMs after differentiation shows the proportion of cTnI-expressing cells.

b, An activation map serving as a visual representation of the activation sequence recorded by means of the MEA data acquisition system. The map activation time (the time duration between the first and last activations) is represented by the lower scale at the bottom of the map. The color strip below the map represents the color spectrum and its scaling according to time. Color coding: red - early; blue - late.

c, A representative display of electrograms recorded from the entire MEA array. Spontaneously beating cardiomyocytes were verified by extracellular electrograms recorded on the 3rd day after cell seeding and culture.

d, MEA micro matrix electrode system to detect the changes of field potential and phase waveform during depolarization/repolarization of hiPS-CMs.

e, Electrical properties of the cells were studied with MEA, which revealed the differences between the standard and compound induced hiPS-CMs group. FPD, field potential duration.

f, Calcium flux in hiPS-CMs at day 15 of the induction. Calcium transients were recorded at the basal condition. Images show traces of calcium transients. Calcium transient frequency at the basal state. Data are presented as the means ± SEM. **P*＜0.05.


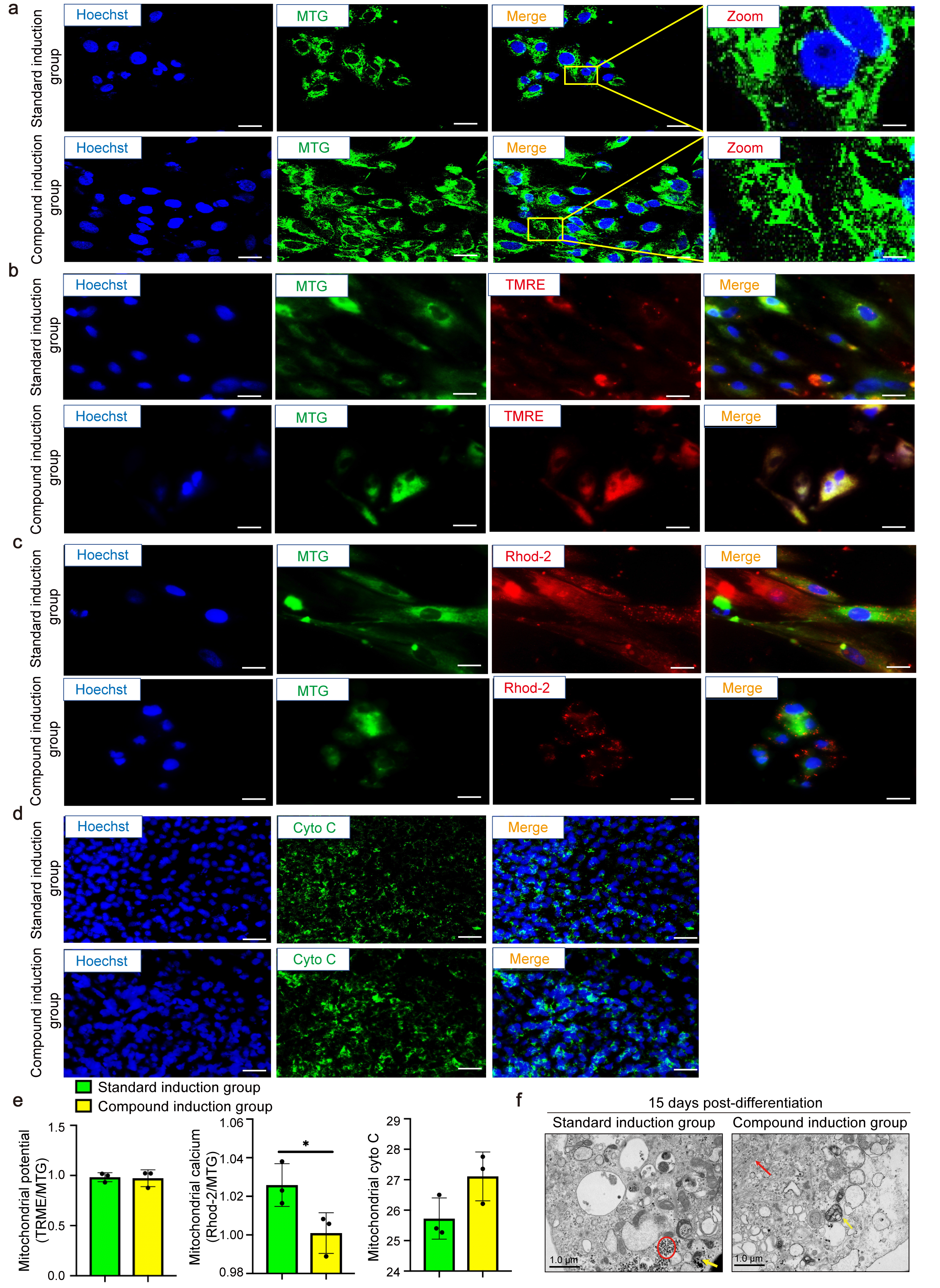


**Figure. S7.**

Comparison of structure and functional maturity of the standard and compound induced hiPS-CMs from hiPSC lines BC (reprogrammed from blood cells).

a, Morphological and structural characteristics of mitochondria in living hiPS-CMs labeled with mitochondrial green fluorescent probe (MTG). Scale bars = 50 μm.

b, The mitochondria were labeled with tetramethylrhodamine methyl ester (TMRE) to detect mitochondrial membrane potential and analyzed with a fluorescence microscope. Scale bars = 50 μm.

c, Detection of mitochondrial calcium level in hiPS-CMs cells by Rhode-2 fluorescence staining. Scale bars = 50 μm.

d, Immunofluorescence staining for cyto C in hiPS-CMs differentiated under the standard and saponin^+^ compound induction protocols. Both groups of cyto C were retained in the mitochondria. Scale bars = 200 μm.

e, The fluorescence intensity of TMRE, Rhod-2 and cyto C staining were quantified using ImageJ, **P* <0.05.

f, Ultrastructural examination of hiPS-CMs myofilament and mitochondria. Red arrow indicates myofilament. Yellow arrows indicate mitochondria. Red circle indicates glycogen accumulation. Scale bars = 1 μm.

**
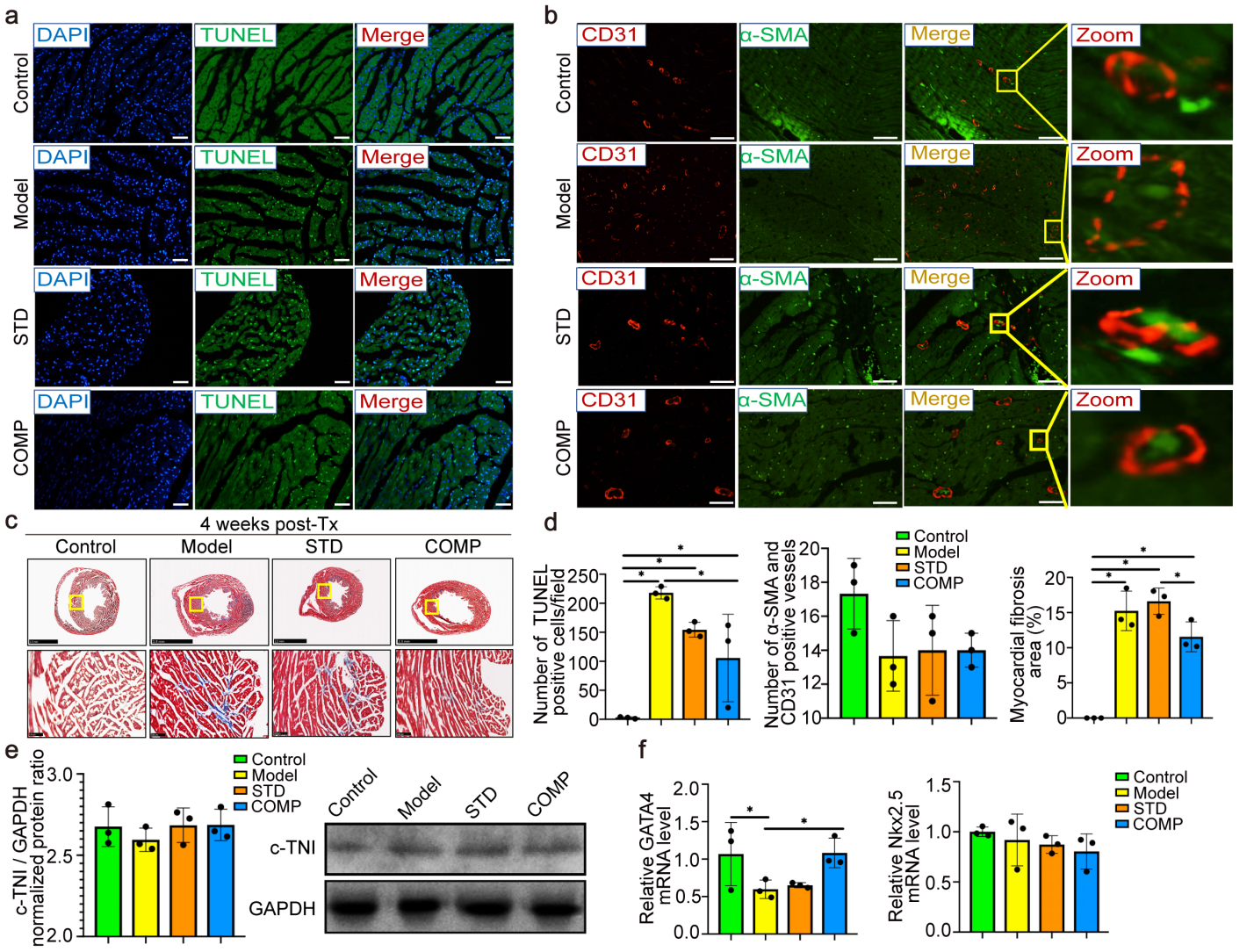
**

**Figure. S8.**

Effects of the compound induced hiPS-CMs on cardiac structure at 4 weeks post transplantation.

a, TUNEL assay was performed to detect the myocardial apoptosis. Apoptotic nuclei were stained green, and normal nuclei were stained blue. Scale bar: 50 μm.

b, Microvascular neo-angiogenesis at 4 weeks post hiPS-CMs transplantation. Immunofluorescent staining for micro-vessels positive for α-smooth muscle actin (α-SMA) and CD31 in the left ventricle. Scale bar: 200 μm.

c, Representative photographs of myocardial fibrosis, which were determined by Masson's trichrome staining. The blue color represented the distribution of collagens.

d, Statistical analysis of myocardial apoptosis, microvascular neo-angiogenesis and fibrosis staining were quantified using ImageJ.

e, The expression of c-TNI protein was analyzed by Western blotting and normalized to GAPDH.

f, Real-time PCR was used to assess cardiac transcription factor GATA4 and Nkx2.5 mRNA levels in the heart. n = 3 mice per group and * indicates that *P*<0.05.

STD, standard induced hiPS-CMs transplantation group; COMP, compound induced hiPS-CMs transplantation group.

**
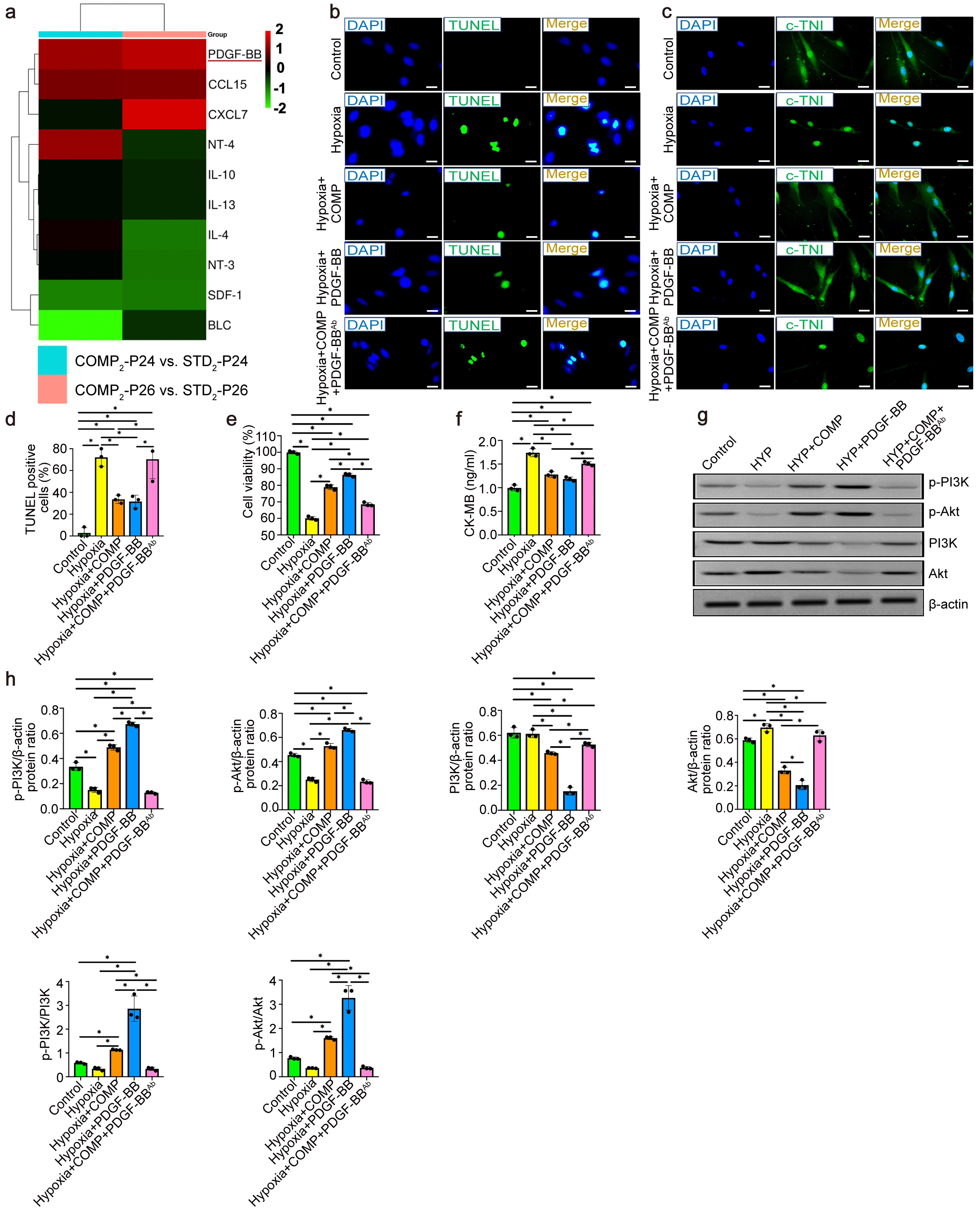
**

**Figure. S9.**

PDGF-BB secreted from the compound induced hiPS-CMs from hiPSC lines BC (reprogrammed from blood cells) rescued the hypoxia-triggered injury model of HCMs by regulating PI3K/Akt pathway *in vitro*.

a, Heat map demonstrated relative expressions of biomarkers from screening cytokine antibody array in the supernatant of the compound and standard induced hiPS-CMs derived from two different generations of hiPS cells (passage 24, 26). The up-regulated cytokines were shown in red and down-regulated cytokines in green.

b, Representative photomicrographs of TUNEL (green) and DAPI (blue) -stained HCMs were shown. Scale bars = 50 μm.

c, Changes of myocardial specific protein c-TNI expression level in hypoxia-injured HCMs was detected by immunofluorescence assay. Scale bars = 50 μm.

d, Quantification of IF staining for TUNEL^+^ HCMs showed that PDGF-BB in the conditioned medium of the compound induced hiPS-CMs was the core cytokine that inhibited the apoptosis of HCMs.

e, Quantitative analysis by CCK-8 assay of cell viability. Values were expressed as the mean ± SD of three independent experiments.

f, The protein expression of CK-MB in the extracellular medium of hypoxia-injured HCMs was detected by ELISA.

g, PI3K, p-PI3K, Akt, p-Akt and β-actin levels of hypoxia-injured HCMs in NC, HYP, HYP+COMP, HYP+PDGF-BB and HYP+COMP+PDGF-BB^Ab^ groups were analyzed by Western blot.

h, The protein expression levels were normalized to those of β-actin. All data shown as mean ± SD, **P*<0.05. HCMs, human cardiac myocytes; HYP: Hypoxia; COMP, the conditioned medium of the compound induced hiPS-CMs; PDGF-BB, platelet-derived-growth-factor-BB; PDGF-BB^Ab^, neutralizing antibody against PDGF-BB; c-TNI: cardiac Troponin I; COMP-P24, the conditioned medium from cardiomyocytes induced by the 24th generation of hiPS by the compound induction scheme; COMP-P26, the conditioned medium from cardiomyocytes induced by the 26th generation of hiPS by the compound induction scheme; STD-P24, the conditioned medium from cardiomyocytes induced by the 24th generation of hiPS by the standard induction scheme; STD-P26, the conditioned medium from cardiomyocytes induced by the 26th generation of hiPS by the standard induction scheme; p-PI3K: phospho-phosphatidylinositol 3-kinase; PI3K: phosphatidylinositol 3-kinase; p-Akt: phospho- protein kinase B; Akt: protein kinase B.

**Table S1.**

Antibodies used in immunofluorescence analyses.

| **Target** | **Host** | **Supplier** | **Cat no.** | **Dilution** |
| --- | --- | --- | --- | --- |
| SMA | Rabbit | Proteintech | 55135-1-AP | 1:200 |
| Cyto C | Mouse | Proteintech | 66264-1-Ig | 1:100 |
| c-TNI | Rabbit | Abcam | Ab52802 | 1:200 |
| CD31 | Rat | Abcam | Ab7388 | 1:100 |
| Donkey anti-Rabbit IgG Alexa Fluor 488 | Donkey | Invitrogen | A21206 | 1:200 |
| Donkey anti-Rabbit IgG Alexa Fluor 647 | Donkey | Invitrogen | A31573 | 1:200 |

SMA: smooth muscle actin; Cyto C: Cytochrome C; c-TNI: cardiac Troponin I.

**Table S2.**

Quantitative PCR primer list.

| **Name** | **Forward primer sequence** | **Reverse primer sequence** |
| --- | --- | --- |
| Human-Nanog | TTTGTGGGCCTGAAGAAAACT | AGGGCTGTCCTGAATAAGCAG |
| Human-Oct3/4 | CTGGGTTGATCCTCGGACCT | CCATCGGAGTTGCTCTCCA |
| Human-Fgf4 | CTCGCCCTTCTTCACCGATG | GTAGGACTCGTAGGCGTTGTA |
| Human-Esg1 | ACTCTCCCGGCACGTAGAC | AGGGATTCGAGATCCGTCCG |
| Human-Dppa2 | GGTGCCAGTTAAAGATGACGC | GAGGCAAAATGGTCGGCAAG |
| Human-Dppa4 | GACCTCCACAGAGAAGTCGAG | TGCCTTTTTCTTAGGGCAGAG |
| Human-actinin | TCCATCGGAGCCGAAGAAATC | GTGTCGGTGGATCAAAGCACA |
| Human-cTNI | TTTGACCTTCGAGGCAAGTTT | CCCGGTTTTCCTTCTCGGTG |
| Human-TNNT2 | GGAGGAGTCCAAACCAAAGCC | TCAAAGTCCACTCTCTCTCCATC |
| Human-Nkx2.5 | GAGCCGAAAAGAAAGCCTGAA | CACCGACACGTCTCACTCAG |
| Human-GATA4 | CGACACCCCAATCTCGATATG | GTTGCACAGATAGTGACCCGT |
| Human-MEF2C | GAACGTAACAGACAGGTGACAT | CGGCTCGTTGTACTCCGTG |
| Human-CX43 | GGTGACTGGAGCGCCTTAG | GCGCACATGAGAGATTGGGA |
| Human-GAPDH | GGAGCGAGATCCCTCCAAAAT | GGCTGTTGTCATACTTCTCATGG |
| Mouse-Nkx2.5 | GACAAAGCCGAGACGGATGG | CTGTCGCTTGCACTTGTAGC |
| Mouse-GATA4 | TCAACCGGCCCCTCATTAAG | GTGGTGGTAGTCTGGCAGT |
| Mouse-GAPDH | AGAAGGCTGGGGCTCATTTG | GTACTCATCATAGGAAACACCA |

**Table S3.**

Antibodies used in western blot analyses.

| **Target** | **Host** | **Supplier** | **Cat no.** | **Dilution** |
| --- | --- | --- | --- | --- |
| c-TNI | Rabbit | Abcam | Ab52802 | 1:1000 |
| p-PI3K | Rabbit | AffinitY | AF3241 | 1:1000 |
| PI3K | Mouse | Proteintech | 60225-1-Ig | 1:5000 |
| p-Akt | Mouse | Proteintech | 66444-1-Ig | 1:2000 |
| Akt | Rabbit | Proteintech | 10176-2-AP | 1:2000 |
| β-actin | Rabbit | Abcam | Ab8227 | 1:2000 |
| Goat Anti-Rabbit IgG H&L (HRP) | Goat | Abcam | Ab205718 | 1:5000 |
| Goat Anti-Mouse IgG H&L (HRP) | Goat | Abcam | Ab205719 | 1:5000 |

c-TNI: cardiac Troponin I; p-PI3K: phospho-phosphatidylinositol 3-kinase; PI3K: phosphatidylinositol 3-kinase; p-Akt: phospho- protein kinase B; Akt: protein kinase B.

**Movie S1.**

Spontaneous pulsation properties of standard induced hiPS-CMs on day 15 of differentiation.

Cell contraction properties of standard induced hiPS-CMs were observed by an inverted microscope. The contraction rate of the spontaneously beating cardiomyocytes was determined by direct visual counting of 30 contractions/min under standard induction condition. It can be seen from the video that the active contraction force of standard induced hiPS-CMs is generally level, and the peripheral cells seem to be driven and pulled by the active beating cells in the middle.

**Movie S2.**

Spontaneous pulsation properties of compound optimally induced hiPS-CMs on day 15 of differentiation.

Change of contractility and frequency of the compound induced hiPS-CMs are clearly observed. The contraction rate of the spontaneously beating cardiomyocytes was evaluated around 90 contractions/min under compound induction condition. It can be seen from the video that the compound induced hiPS-CMs beat in sheets and waves, and almost all of them can actively beat with strong contractions, and the beating state is very similar to that of normal human cardiomyocytes.
