# Supplementary figures and images for: Cardiomyocytes induced from hiPSCs by well-defined compounds have therapeutic potential in heart failure by secreting PDGF-BB
Source: Signal Transduct Target Ther. 2022 Jul 29;7:253. doi: 10.1038/s41392-022-01045-4 (PMC9334380; doi:10.1038/s41392-022-01045-4)

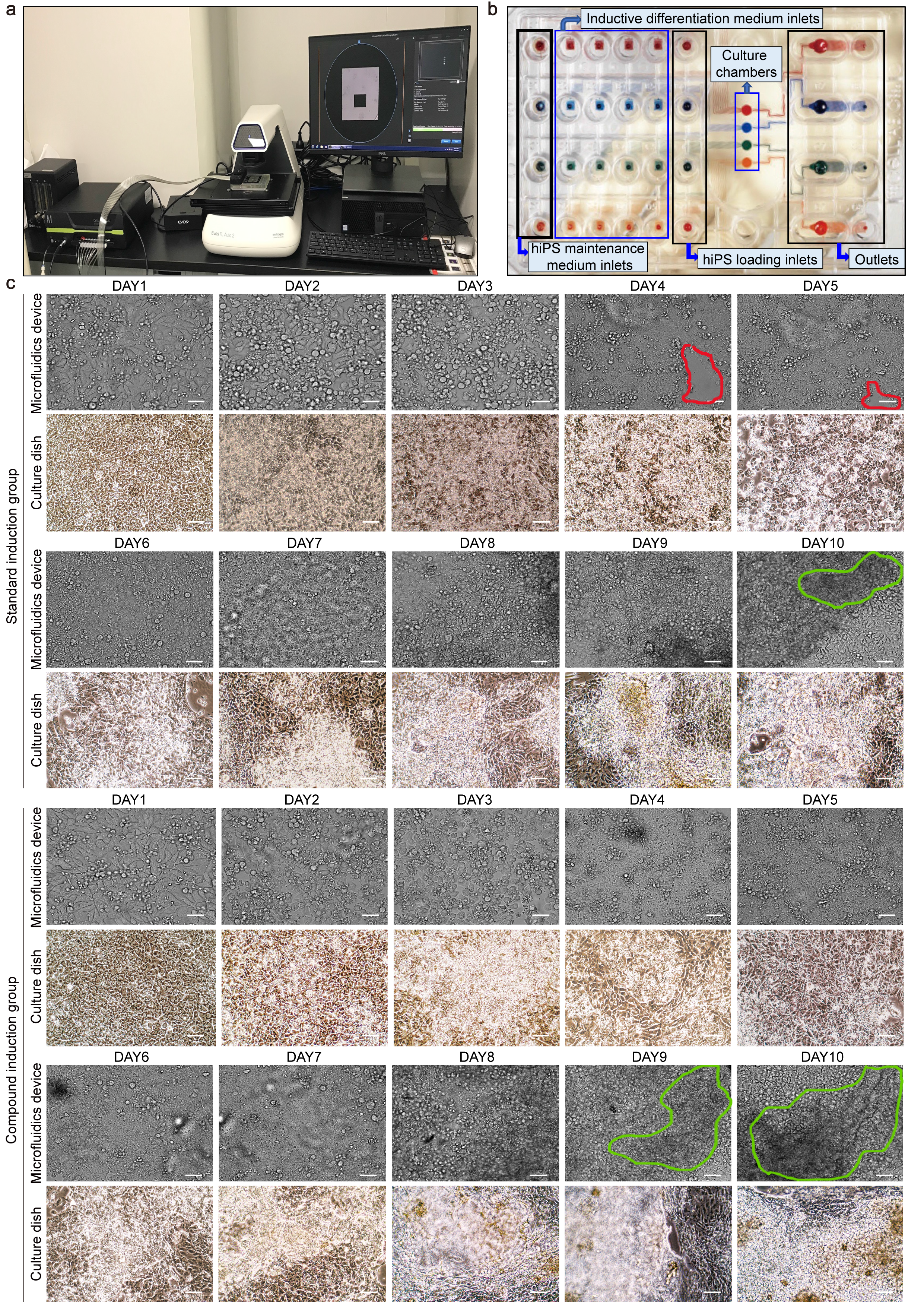

Supplement: Supplementary file 2 — Supplementary figure 3 [file 41392_2022_1045_MOESM2_ESM.tif]
